# Spatial Metabolomics Reveals Microregion-Specific Neurochemical Perturbations in the Brains of PCPA-Induced Insomniac Rats: Integration of MALDI-MSI and Targeted LC-MS/MS

**DOI:** 10.3390/metabo16080543

**Published:** 2026-07-31

**Authors:** Yan Yan, Jiaying Liu, Yingjian Deng, Yu Tao, Xinxin Li, Chenhui Du, Kun Yang, Ruiping Zhang

**Affiliations:** 1 Shanxi Bethune Hospital, Shanxi Academy of Medical Sciences, Third Hospital of Shanxi Medical University, Tongji Shanxi Hospital, Taiyuan 030032, China; yanyan520@sxu.edu.cn; 2 Modern Research Center for Traditional Chinese Medicine, Shanxi University, Taiyuan 030006, China; liujiaying1@sxu.edu.cn (J.L.); dengyingjian@sxu.edu.cn (Y.D.); taoyu0516@163.com (Y.T.); lixinxin0830@163.com (X.L.); 3 School of Traditional Chinese Materia Medica, Shanxi University of Chinese Medicine, Taiyuan 030619, China; dch@sxtcm.edu.cn; 4 The Radiology Department, Shanxi Provincial People’s Hospital Affiliated to Shanxi Medical University, Taiyuan 030001, China

**Keywords:** mass spectrometry imaging, derivatization, spatial metabolomics, insomnia, LC-MS/MS

## Abstract

**Objectives:** Insomnia is a highly prevalent sleep disorder involving complex neurochem-ical dysregulation; however, the spatial distribution of neurotransmitters and small-molecule metabolites across distinct brain microregions in the insomniac state re-mains poorly characterized. This study aimed to map region-specific metabolic perturba-tions in situ in a p-chlorophenylalanine (PCPA)-induced insomnia rat model using opti-mized matrix-assisted laser desorption/ionization mass spectrometry imaging (MAL-DI-MSI) integrated with targeted metabolomics validation. **Methods:** On-tissue chemical derivatization MALDI-MSI was optimized using α-cyano-4-hydroxycinnamic acid (CHCA) as the matrix on a Bruker tims TOF flex mass spectrometer. The rats received PCPA (400 mg/kg, intraperitoneal) for three days to establish the insomnia model. Metabolite identifi-cation was conducted using MetaboScape® software (2020b) and the Human Metabolome Database. Ultra-performance liquid chromatography–tandem mass spectrometry was em-ployed to quantify nine key neurotransmitters and metabolites across six brain microre-gions. Key synthetic enzyme expression was evaluated by immunofluorescence and West-ern blotting analyses. **Results:** TMP-TFB-derived brain slices clearly showed the distribu-tion of neurotransmitters in brain microregions. MALDI-MSI demonstrated that the spatial distribution and abundance of eight neurotransmitters were disturbed in brains of PCPA-induced insomniac rats. A total of 346 metabolites were characterized across six brain microregions (cerebellum, cortex, hippocampus, brainstem, hypothalamus, and stri-atum), with principal coordinate analysis revealing clear metabolic separation between control and insomniac rats. PCPA treatment markedly disrupted tryptophan and tyrosine metabolism, evidenced by decreased 5-HT, 5-HTP, and 5-HIAA, alongside region-specific alterations in DA, NE, HVA, and Ach. Additionally, GABA levels decreased in the hippo-campus, striatum, and hypothalamus, whereas glutamate increased throughout the brain. Targeted metabolomics validated the MSI findings, and Bland–Altman analysis confirmed good consistency between the two analytical platforms. PCPA further disturbed the meta-bolic enzymes MAOA, DDC, and TPH2 within the Trp-5-HTP-5-HT-5-HIAA metabolic pathway in the brainstem and TYH and DBA within the tyrosine-DA-NE metabolic pathway in the striatum. **Conclusions:** This study demonstrates that region-specific altera-tions in tryptophan and tyrosine metabolism pathways provide mechanistic insights into insomnia pathogenesis and potential therapeutic targets.

## 1. Introduction

Insufficient sleep is a significant public health issue, affecting around 10% of adults with insomnia [1]. Chronic sleep disruption has been associated with immunological and cognitive dysfunctions and metabolic conditions [2]. Over the last ten years, various observational studies have linked chronic insomnia to an increased risk of cardiometabolic diseases, including type 2 diabetes and cardiovascular diseases [3,4]. The brain plays a central role in regulating metabolic physiology and cognitive functions. Insomnia is widely believed to be linked to neurotransmitter disturbances in the brain, as highlighted by its complex pathogenesis. Key neurotransmitters and neuromodulators, including acetylcholine (Ach), dopamine (DOPA), glutamate (Glu), histamine (His), hypocretin, and norepinephrine (NE), play crucial roles in promoting wakefulness [5]. Various brain regions work together to influence sleep and wakefulness, with specific neuronal groups producing different neurotransmitters that hierarchically control sleep–wake cycles [6].

Numerous analytical technologies have elucidated the tissue-specific distributions of various compounds. Nuclear magnetic resonance (NMR) and mass spectrometry (MS) are extensively utilized analytical techniques for examining small-molecule metabolites linked to neurological diseases, including Alzheimer’s and Parkinson’s [7]. ^1^H-magnetic resonance spectroscopy (^1^H-MRS) offers non-invasive detection and quantification of key brain metabolites, such as N-acetylaspartate, choline, creatine, gamma-aminobutyric acid (GABA), and myoinositol [8]. The spatial distribution and modification of metabolites in tissue are frequently unreported, as analyses typically use tissue homogenates, leading to a loss of spatial information relevant to metabolites. Specific brain regions are thought to regulate wakefulness and sleep by forming neural circuits [9]. Investigating the tissue-specific distribution and regional changes in various brain molecules is essential to understanding their roles in insomnia development.

Matrix-assisted laser desorption/ionization mass spectrometry imaging (MALDI-MSI) facilitates the in situ visualization of the abundance and spatial distribution of endogenous lipids, peptides, small proteins, and metabolites across diverse samples [10]. MSI offers label-free, precise chemical specificity and effective tissue imaging capabilities and can map the distribution of small metabolites, lipids, glycans, proteins, and peptides without prior knowledge of the target molecules [11]. It is regarded as an ideal tool for spatial metabolomics because it offers both molecular and spatial data concurrently. It has been extensively used to investigate the molecular pathways of neurodegenerative diseases, such as Parkinson’s and Alzheimer’s, in animal models, as well as the effects of the traditional Chinese medicines Stephania tetrandra and Polygala tenuifolia [12].

To explore metabolic changes in various brain regions following p-Chlorophenylalanine (PCPA) administration, MALDI MSI matrix was optimized for visualizing regional metabolic coverage and ion abundance related to neurotransmitter alterations. MSI was used to identify the spatial distribution of and variations in NTs. We developed a targeted metabolomics analysis method to compare the MSI results. Immunofluorescence and Western blotting analyses revealed the underlying molecular mechanisms.

## 2. Materials and Methods

### 2.1. Chemicals and Reagents

Mass spectrometric-grade acetonitrile (ACN), methanol (MeOH) and trifluoroacetic acid (TFA) were purchased from Thermo Fisher Scientific Co., Ltd. (Waltham, MA, USA). PCPA was obtained from Tokyo Chemical Industry Co., Ltd., Shanghai, China. Serotonin (5-HT) (No. J2123590), GABA (No. H1829037), Glu (No. J2128546), 5-hydroxyindole-3-acetic acid (5-HIAA) (No. REMPO-RA), homovanillic acid (HVA) (No. S07J6G1), tyramine (No. H1622034) and NE (No. H2126349) were obtained from Aladdin Biochemical Technology Co., Ltd., Shanghai, China. Ach (51-84-3) was procured from the National Institute for Drug Standard Material Inspection in Beijing, China. 5-Hydroxytryptophan (5-HTP) (No. ERMPO-BK), glutamine (No. FD7IB206518) carnitine hydrochloride (No. H24S6Y3559), adenosine (No. J08HB173656), L-tryptophan-d3 (No. C2421293), alanine (No. S04IB224798), lysine (No. 804B022), glycine (No. 625A024) and arginine (No. 412B021) were purchased from Yuanye Bio-Technology Co., Ltd., Shanghai, China. Tyrosine (BCBG4812V) was obtained from Sigma-Aldrich, Ltd., Shanghai, China. The purity of all these references was higher than 99%.

Matrix preparation materials, α-Cyano-4-hydroxycinnamic acid (CHCA) and 2,5-dihydroxybenzoic acid (DHB), were procured from Sigma-Aldrich in Shanghai, China. The derivatization reagent 2,4,6-trimethylpyrylium tetrafluoroborate (TMP-TFB) was sourced from J&K Scientific in Beijing, China, while 2,4,6-triphenylpyrylium tetrafluoroborate (TPP-TFB) and 4-hydroxy-3-methoxycinnamaldehyde (CA) were acquired from Sigma-Aldrich in Shanghai, China. The PVC embedding box was acquired from CITOTEST Labware Manufacturing Co., Ltd. (Nantong, China).

### 2.2. Animals

Male Sprague–Dawley rats (250 ± 20 g) were acquired from Beijing Vital River Experimental Animal Co., Ltd., Beijing, China (SCXK 2021-0011). The rats acclimated to laboratory conditions (22 ± 2°C temperature, 55 ± 5% humidity, 12/12 h light/dark cycle) for 7 days before being randomly assigned to either the control group (CN) or the model group (MD). Laboratory food and water were provided without restriction. Rats in the MD group were administered intraperitoneal injections of PCPA in a 2% tween-80 solution at 400 mg/kg daily for three days. Based on our research method, LC-MS/MS was used to determine the serum 5-HT content after three days of PCPA injection [13]. The 5-HT levels in the MD group (90.35 ± 5.02 ng/mL) were significantly lower than those in the CN group (149.21 ± 8.97 ng/mL, *p* < 0.001), indicating the successful creation of the PCPA-induced insomnia rat model. Behavioral experiment results are presented in Figure S1. Three days later, rats were anesthetized using isoflurane, followed by blood collection from the abdominal aorta. The brain tissues were immediately removed after the rats were sacrificed. A total of three rats were used within each group for MALDI-MSI analysis (*n* = 3). All procedures followed the Shanxi University Guidelines for the Care and Use of Laboratory Animals (approval number: SXULL 2022029; date: 15 March 2022).

### 2.3. Optimization of Matrices for Target Plate Determination

Before the on-tissue chemical derivatization experiment, the matrix was optimized using the target plate. After derivatization, the MALDI matrix (DHB or CHCA) was applied to the target plates. DHB (10 mg/mL) was prepared in methanol/H_2_O (70:30, *v*/*v*) containing 4% TFA, and CHCA (10 mg/mL) was prepared in acetonitrile/H_2_O (70:30, *v*/*v*) containing 4% TFA. Initially, 1 μL of 1 μg/μL solutions of 5-HT, GABA, Glu, Ach, DA, NE, 5-HIAA, and HVA, each dissolved in methanol, was applied to a stainless-steel sample plate. Subsequently, 1 μL of a CA derivatization reagent (2 mg/mL in MeOH) was added, and the mixture was allowed to dry at room temperature. Finally, the plate was coated with 1 μL of either DHB or CHCA (10 mg/mL).

### 2.4. Sample Preparation for MALDI MSI Analysis

Rat brains were rapidly collected on an ice platform, rinsed with ice-cold saline to eliminate blood contamination, and dried with filter paper to remove excess saline. The brain tissue was embedded in a 3% CMC-Na solution and stored at −80 °C until needed. Slides of 12 μm thickness and slides of 5 μm thickness were thaw-mounted onto adhesive slides. The harvested 5 μm adhesive slides were fixed in 4% PFA for H&E staining. Twelve-micrometer sagittal sections of frozen cerebrum were prepared using a Leica 3050S cryostat (Leica Biosystems, Nussloch, Germany) at −23 °C and mounted on conductive indium tin oxide (ITO)-coated glass slides.

### 2.5. Optimization of In Situ Brain Tissue Derivation Regents and Spraying Method

For on-tissue derivatization, brain tissue sections were sprayed with CA (2 mg/mL in methanol), TPP-TFB (3 mg/mL in 75% methanol), and TMP-TFB (4 mg/mL in 75% methanol) using an automatic HTX^TM^-Sprayer for comparative analysis. Then, the slides were incubated in a chamber containing saturated vapor of a 50% methanol solution at room temperature for 30 min. Following incubation, the slides were transferred to a vacuum desiccator to eliminate condensed water before matrix deposition. Subsequently, tissue sections were coated with CHCA (10 mg/mL in 70% acetonitrile with 4% TFA) using the HTX^TM^-Sprayer, a fully automated matrix sprayer (Chapel Hill, NC, USA). The spraying parameters were as follows: flow rate of 0.1 mL/min, a total of 10 sprays, nozzle temperature set at 75 °C, nozzle movement speed of 1200 mm/min, nozzle line spacing of 3 mm, a distance of 3 cm between the nozzle and the sample, a total of 30 spray cycles, and nitrogen pressure of 10 psi.

### 2.6. MALDI MS and MSI Experiments

MALDI MS and MSI were performed using a tims TOF flex-MALDI 2 (Bruker, Billerica, Germany) equipped with a Smartbeam 3D laser (λ = 355 nm). Tissue sections underwent analysis in both positive and negative reflectron ion modes, utilizing 100 laser shots at a frequency of 1000 Hz and 63% laser energy, with imaging conducted at a 100 μm laser step size. MALDI MS spectra were collected within an *m*/*z* range of 50 to 800. Data acquisition and export were performed using FlexImaging software (v 4.0) (Bruker, Billerica, Germany). A MALDI tims TOF flex high-resolution apparatus was employed for precise *m*/*z* assignment. The identification of unknown compounds in the MSI data was conducted using MetaboScape^®^ software 2020a (Bruker, Billerica, Germany), the online Human Metabolomics Database (HMDB, https://www.hmdb.ca) and LIPID MAPS (https://www.lipidmaps.org/).

### 2.7. Targeted Metabolomics via LC-MS/MS Experiments

UPLC-MS/MS was employed for quantitation of metabolites identified across six brain microregions to validate the MALDI MSI findings. Briefly, 20 mg cortex tissue samples were weighed and homogenized in 200 μL of physiological saline. Then 150 μL of the supernatant was spiked with 10 μL of L-tryptophan-d3 and 600 µL of ACN, followed by vortexing for 5 min, and centrifuged at 16,060 *g* for 15 min at 4 °C. The supernatants were quantitatively transferred and evaporated to dryness at 37 °C under a gentle nitrogen stream. The residue was redissolved in 200 μL of the initial mobile phase, and a 3 μL aliquot was injected into the LC-MS/MS system for analysis. Supplementary File and Table S1 detail the method and MRM parameters.

### 2.8. Immunofluorescence Staining

Immunofluorescence staining was performed on 5 μm adhesive slides as previously described. After rewarming, the slides were immersed in 0.5% Triton X-100 in PBS for 15 min and then blocked with a 5% BSA solution for 1 h at room temperature. Then the slices were incubated with primary antibodies applied in 3% BSA, including rabbit anti-DDC (1:400, Proteintech, 10166-1-AP, Wuhan, China), rabbit anti-TPH2 (1:100, Abmart, PH9000M, Shanghai, China), rabbit anti-monoamine oxidase A (MAOA) (1:50, Abmart, PK95414, Shanghai, China), tyrosine hydroxylase (TYH) (1:100, A12756, ABclonal, Woburn, MA, USA), and dopamine beta-hydroxylase (DBH) (1:100, A99284, Nature Biosciences, Beijing, China), overnight at 4 °C. On the second day, the slides underwent three washes with PBST, followed by a 2 h incubation with goat anti-rabbit IgG (H+L)-Alexa Fluor^®^ 488 (1:400, Servicebio, GB25303, Wuhan, China). Subsequently, the slides were washed three times with PBS. Subsequently, 4′, 6-diamidino-2-phenylindole (DAPI) staining was conducted by incubating the sample for 5 min in a 1:1000 DAPI solution, followed by washing and sealing for storage. Immunofluorescence images were captured with an EVOS^TM^ M5000 inverted fluorescence microscope (Thermo Fisher Scientific, Waltham, MA, USA). Image processing and fluorescence intensity analysis were performed with ImageJ 1.54f software (NIH, Bethesda, Montgomery County, MD, USA).

### 2.9. Western Blotting

Brainstem and striatum tissue samples were extracted using RIPA buffer (Solarbio Life Sciences, Beijing, China) with Phenylmethanesulfonyl fluoride protease inhibitor (Servicebio, Wuhan, China). Protein concentration was measured post-extraction using a bicinchoninic acid (BCA) protein assay kit (Beyotime, Shanghai, China). Samples containing 25 μg of total protein each were subjected to SDS-PAGE, initially at 60 V for 30 min, then at 80 V for 1 h, and finally at 120 V for 30 min. The proteins were transferred onto 0.45 μm PVDF membranes using a wet transfer system at 300 mA for 40 min. The membranes were blocked with 5% non-fat milk in TBST at room temperature for 2 h, followed by incubation with primary antibodies at 4 °C for 16 h. The following day, the membranes were washed six times with TBST (5 min each wash) and then incubated with the corresponding horseradish peroxidase (HRP)-conjugated secondary antibody at room temperature for 2 h. Protein bands were visualized using an imaging system (Universal Hood II, Bio-Rad, Hercules, CA, USA) with enhanced chemiluminescence (ECL) reagents. Band intensities were normalized to GAPDH as a loading control and quantified with ImageJ software (NIH, Bethesda, MD, USA).

The primary antibodies used in this study were as follows: DDC (1:1000, 10166-1-AP, Proteintech), TPH2 (1:500, PH9000S, Abmart), MAOA (1:1000, PK95414S, Abmart), TYH (1:1000, A12756, AB clonal), DBH (1:1500, A99284, Nature Biosciences) and glyceraldehyde-3-phosphate dehydrogenase (GAPDH) (1:5000, GB15004-100, Servicebio). The secondary antibody used was HRP-conjugated goat anti-rabbit IgG at a dilution of 1:8000 (bs-G-HRP, Bioss, Beijing, China).

### 2.10. Data Analysis

Principal component analysis (PCA) and volcano plot analysis were employed to statistically assess potential metabolite changes. Pathway analysis was conducted using MetaboAnalyst 6.0 online (www.metaboanalyst.ca, accessed on 9 January 2025). Pathway mapping for MALDI-MSI data utilized the KEGG pathway database (www.genome.jp, accessed on 16 January 2025).

## 3. Results

### 3.1. Optimization of DHB and CHCA as Matrices for MALDI-MSI

**Figure 1:**
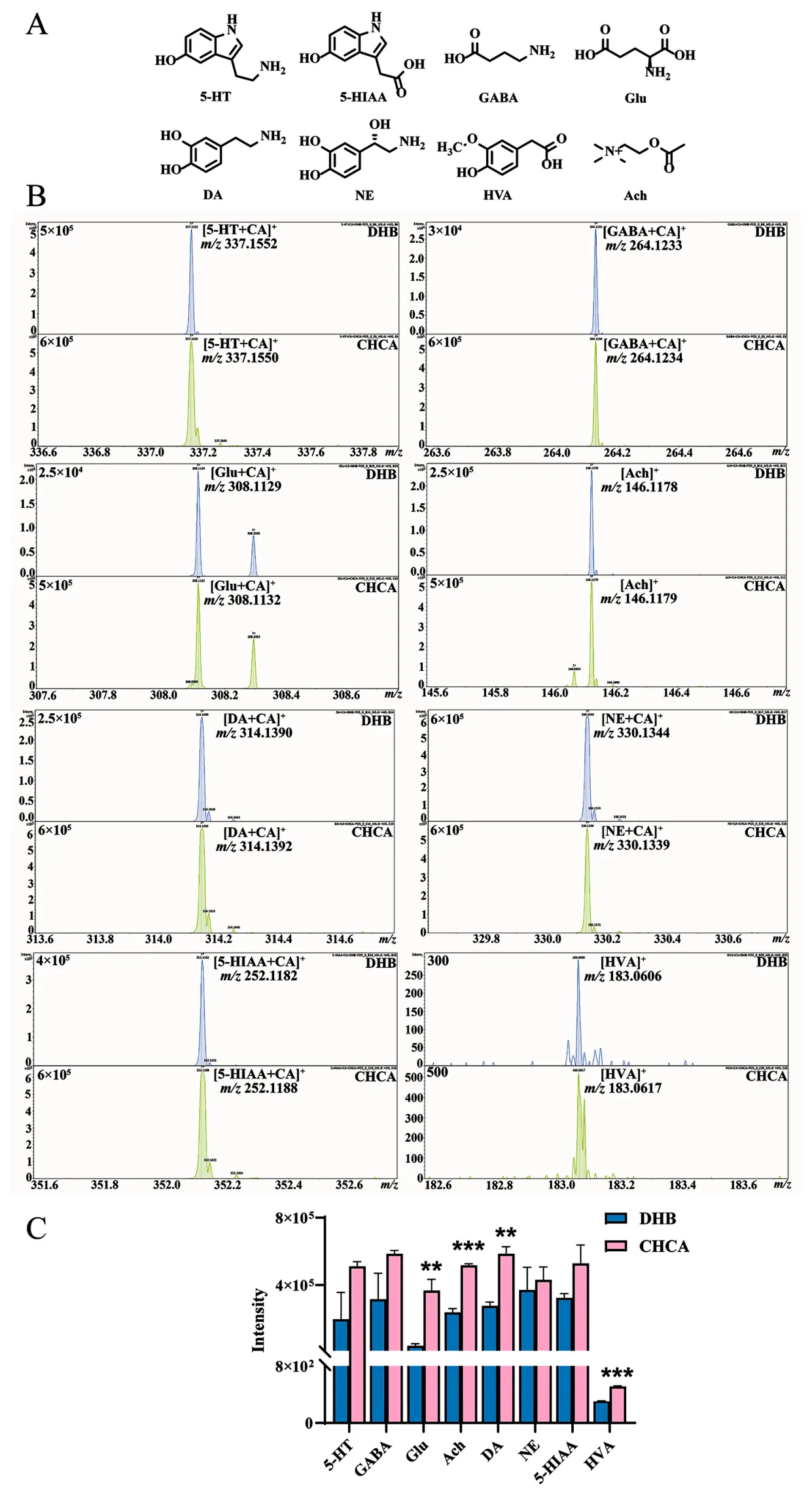
(**A**). Chemical structures of 8 neurotransmitters. (**B**). Mass spectra of 8 neurotransmitters with DHB and CHCA as matrices. (**C**). Mass spectra analysis of 8 neurotransmitters using DHB or CHCA as a matrix revealed intensity measurements (*n* = 3) for 5-HT, GABA, Glu, Ach, DA, NE, 5-HIAA, and HVA. Statistical significance is denoted by ** *p* < 0.01, and *** *p* < 0.001 compared to DHB.

**Figure 2:**
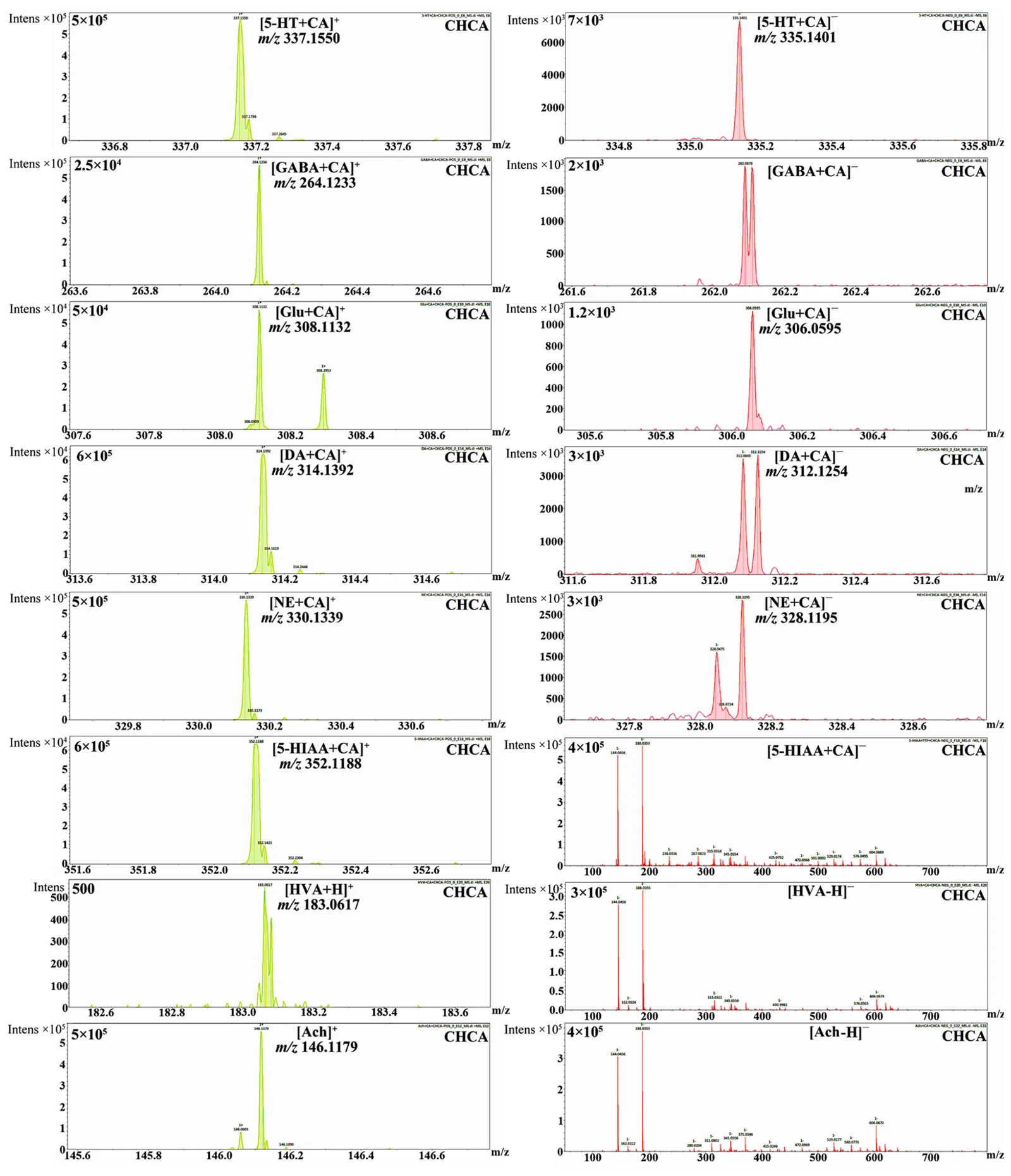
The mass spectra of eight neurotransmitters in both positive (green) and negative (red) ion modes using CHCA as the matrix within the *m*/*z* range of 50–800.

In this study, eight neurotransmitters (5-HT, GABA, Glu, Ach, DA, NE, 5-HIAA and HVA) were derivatized using CA (Figure 1A), and then two different matrices, DHB and CHCA, were compared. In positive ion mode, derivatized compounds such as 5-HT (337.1550), 5-HIAA (352.1184), Ach (146.1179), DA (314.1392), Glu (308.1132), and GABA (264.1234) were detected using DHB or CHCA (Figure 1B). However, CHCA exhibited superior performance in terms of the detection intensity of Glu, Ach, DA and HVA (Figure 1C). The chemical structures of Ach and HVA were unable to react with CA, so they were detected with lower intensity. In positive ion mode, CHCA demonstrated enhanced detection sensitivity and broader molecular coverage within the 50–800 *m*/*z* range (Figure 2). The intensities of [5-HT+CA]^−^, [GABA+CA]^−^, [Glu+CA]^−^, [DA+CA]^−^, and [NE+CA]^−^ were lower in the negative ion mode (intensity 10^^3^) compared to the positive ion mode. The results indicated that CHCA exhibited higher signal intensities for derivatized neurotransmitters, while both CHCA and DHB matrices produced a similar number of matrix interference peaks.

### 3.2. Optimization of Derivatization Reagents and Matrix Spraying Method In Situ in Brain Tissue

**Figure 3:**
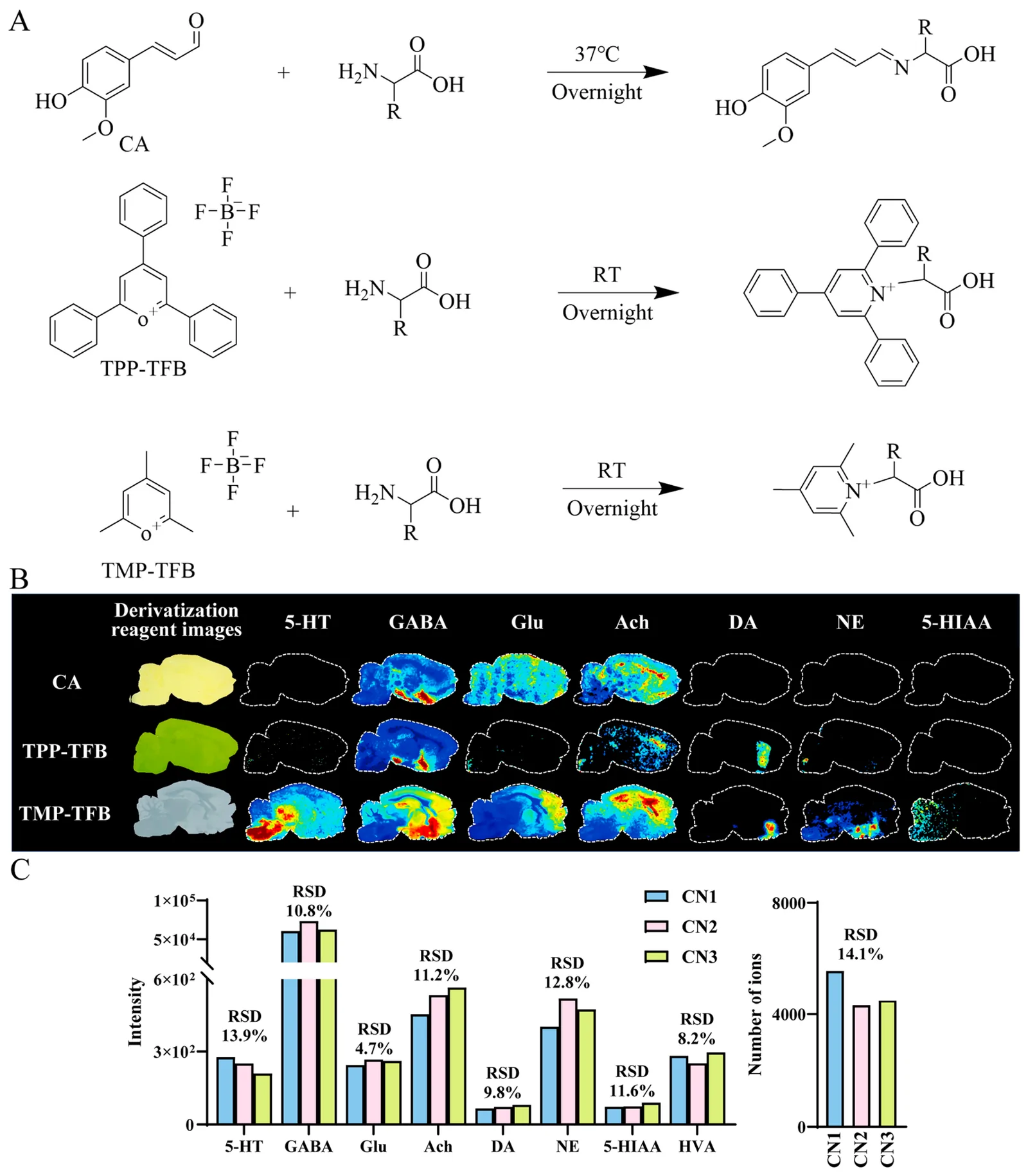
(**A**). Schematic derivatization reaction of an amino acid with CA, TPP-TFB and TMP-TFB. (**B**). MSI visualizations of neurotransmitter derivatives using CA, TPP-TFB, and TMP-TFB in sagittal sections of rat brains. (**C**). Repeatability of MSI peak intensities of 8 neurotransmitters in brain slices from three normal rats and repeatability of the number of ions detected by MSI in brain slices from three normal rats (*n* = 3).

Detecting amino metabolites and neurotransmitters in tissue via MALDI-MSI is challenging due to low ionization efficiency and interference from matrix background peaks [14]. We optimized the derivatization reaction conditions to enhance detection sensitivity while preserving the spatial distribution of metabolites in tissue. Firstly, three commercially available derivatization reagents, CA, TPP-TFB and TMP-TFB, were applied to the CHCA-coated tissues for imaging analysis (Figure 3A). As shown in Figure 3B, the optical images show different colors for the three different derivatization reagents. As we expected, 5-HT, GABA, Glu, Ach, DA, NE and 5-HIAA were all detected using TMP-TFB. However, only three (GABA, Glu and Ach) and five (GABA, Glu, Ach, DA and NE) were detected when using CA and TPP-TFB, respectively. More specifically, TMP-TFB-derived brain slices clearly showed the distribution of neurotransmitters in brain microregions, and the sensitivity was higher than that of CA and TPP-TFB. Then, we optimized the matrix spraying method. Among three different conditions (A, B and C), MSI profiles under condition C (10 sprays, 30 s drying time) exhibited improved clarity and more distinct microregion delineation than those under conditions A and B at a spatial resolution of 100 μm (Figure S2A,B). 

### 3.3. Investigation of Stability of the Quality Axis and Repeatability of the Method

MALDI-MSI usually requires continuous scanning for a relatively long time. For TOF mass spectrometers, this might cause a stability drift of the instrument’s mass axis [15]. The study utilized a 100 μm spatial resolution for MSI analysis, resulting in a relatively extended scanning duration. Using the SCiLS^TM^ Lab 2022b software, the data of 8 neurotransmitter ion peaks from different regions of the same brain tissue section were extracted for statistical analysis. The findings indicated that the mass precision fluctuations in the *m*/*z* values for the eight neurotransmitters remained within ±10 ppm (Table S2), demonstrating the instrument’s stable mass axis and ensuring reliable long-term continuous scanning.

Furthermore, three normal rat brain sections were selected for MALDI-MSI analysis to examine the reproducibility of the method. Statistical analysis was performed on the intensities of eight neurotransmitter ion peaks, ensuring a signal-to-noise ratio (S/N) exceeding 50.0. As shown in Figure 3C, the RSD values of the ion peak intensity and quantity of the eight neurotransmitters in the brain sections of the three normal rats were all less than 15.0%. These results indicated that this method had good repeatability.

### 3.4. Revealing Regional Alterations in Neurotransmitters in PCPA-Induced Rat Brains Using MALDI MSI with CHCA

**Figure 4:**
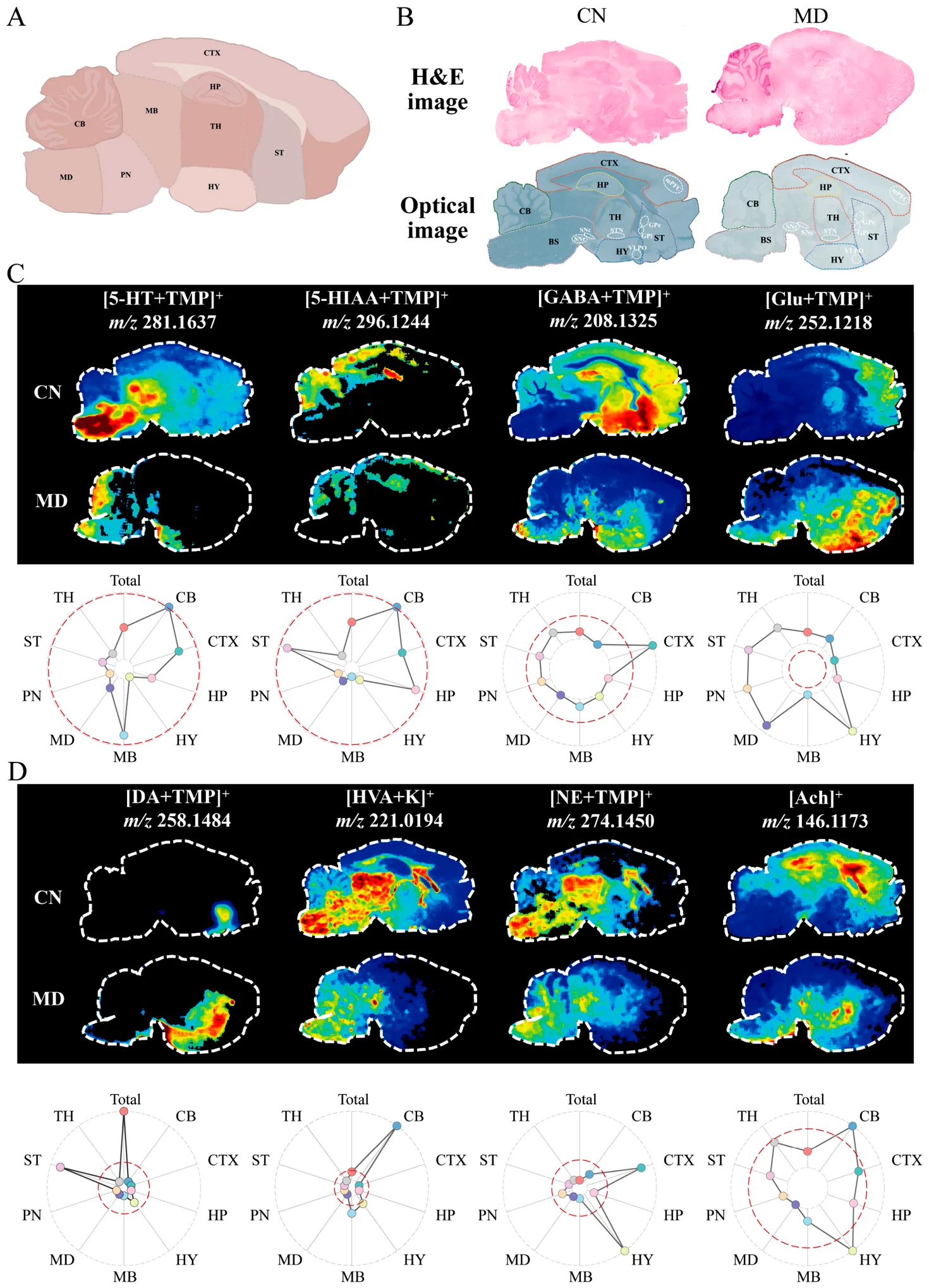
Visualization of neurotransmitters in rat brains detected using MALDI MSI. (**A**) Microregions of rat brain. CB: cerebellum; CTX: cerebral cortex; HP: hippocampus; HY: hypothalamus; MB: middle brain; and ST: striatum. (**B**) H&E staining and optical images of brain sections. (**C**,**D**) Spatial distributions of and changes in neurotransmitters 5-HT, GABA, Glu, Ach, DA, NE, 5-HIAA and HVA in CN group and MD group (*n* = 3). Each dot represents the FC value of the metabolite in this brain region. The red dashed line indicates FC=1.

A key challenge in pathological studies is identifying metabolites that may be related to disease. We employed a PCPA-induced rat model of insomnia to assess the proposed method and visualize alterations in eight key neurotransmitters within normal and MD sagittal brain tissue sections. Based on sagittal section H&E staining and optical images, nine brain microregions were identified as regions of interest (ROIs) due to their close association with sleep–wake cycles: cerebellum (CB), cerebral cortex (CTX), hippocampus (HP), hypothalamus (HY), midbrain (MB), medulla (MD), pons (PN), striatum (ST), and thalamus (TH), as illustrated in Figure 4A,B. As shown in Figure 4C, derivatized 5-HT (*m*/*z* 281.1637) was mainly localized in the brainstem (PN, MD and MB), CB, and HY. Its downstream metabolite 5-HIAA (*m*/*z* 296.1244) was mainly distributed in the CB, CTX and HP. Previous MSI studies reported that 5-HT was localized in the HY and the MB area [16]. Källback et al. [3] found that 5-HIAA was predominantly located in the HP and ST regions. This study demonstrated a significant reduction in the relative abundances of 5-HT in the brainstem and 5-HIAA in the CB, CTX, and HP of MD group rats compared to those in the CN group.

GABA, a key inhibitory neurotransmitter in the brain, promotes sleep, and increasing its levels can alleviate anxiety [17]. Glu, recognized for its role in sleep suppression, influences the central nervous system (CNS) via two main receptor types: ionotropic and metabotropic Glu receptors. This study identified derivatized GABA (*m*/*z* 208.1325) as the most abundant compound in the HY, ST, and MB regions of sagittal rat brain film, consistent with findings by Liu et al. (2022) in their metabolomics study [18]. We also found that PCPA treatment notably reduced GABA levels in the HP, ST, and HY regions while elevating Glu levels across the brain, aligning with findings by Yan et al. [19,20].

Moreover, we identified neurotransmitters and metabolites from catecholaminergic neurons, such as DA, NE, and HVA. These neurotransmitters are collectively known as catecholamines and play important roles in the sleep–wake process [21]. Our study identified derivatized DA (*m*/*z* 258.1484) primarily in the ST region of CN rat brain tissue, attributed to its dense dopaminergic innervation from the substantia nigra [6,22]. As shown in Figure 4D, MD group rats showed a higher intensity of DA in the ST region. In addition, we also detected the downstream metabolite of DA, that is, the ion corresponding to derivatized HVA. In contrast, the relative abundances of HVA and NE were increased, while the relative abundance of Ach (*m*/*z* 146.1173) in the brainstem showed a downward trend following PCPA injection. Ach is a neurotransmitter and neuromodulator involved in a variety of cognitive functions and REM sleep [23]. The results demonstrated that the spatial distributions and abundances of these neurotransmitters were disturbed in PCPA-induced insomniac rats.

### 3.5. Global Spatial Metabolic Profiling in PCPA-Induced Insomniac Rat Brains

An unsupervised PCA model based on MS image pixel points was developed to identify global discriminating molecules between insomnia and normal rats in the brain, aiming to screen region-specific biomarkers. First, the coverage degree of metabolites was assessed. In the positive ion mode, as shown in Figure S3A, 5559 ion peaks were detected with a signal-to-noise ratio exceeding 50, and 346 ion peaks were characterized as metabolites using MetaboScape^®^ software. As shown in Figure S3B, the *m*/*z* range of 50–400 mainly included derivatized amino acids, nucleotide metabolites, and fatty acids, while the *m*/*z* range of 400–800 mainly included lysophospholipids (LPC), lyso-phosphatidylethanolamine (LPE), Phosphatidylcholine (PC), phosphatidylethanolamine (PE), phosphatidic acid (PA), ceramide (Cer), phosphatidylserine (PS), sphingolipid (SM), etc.

**Figure 5:**
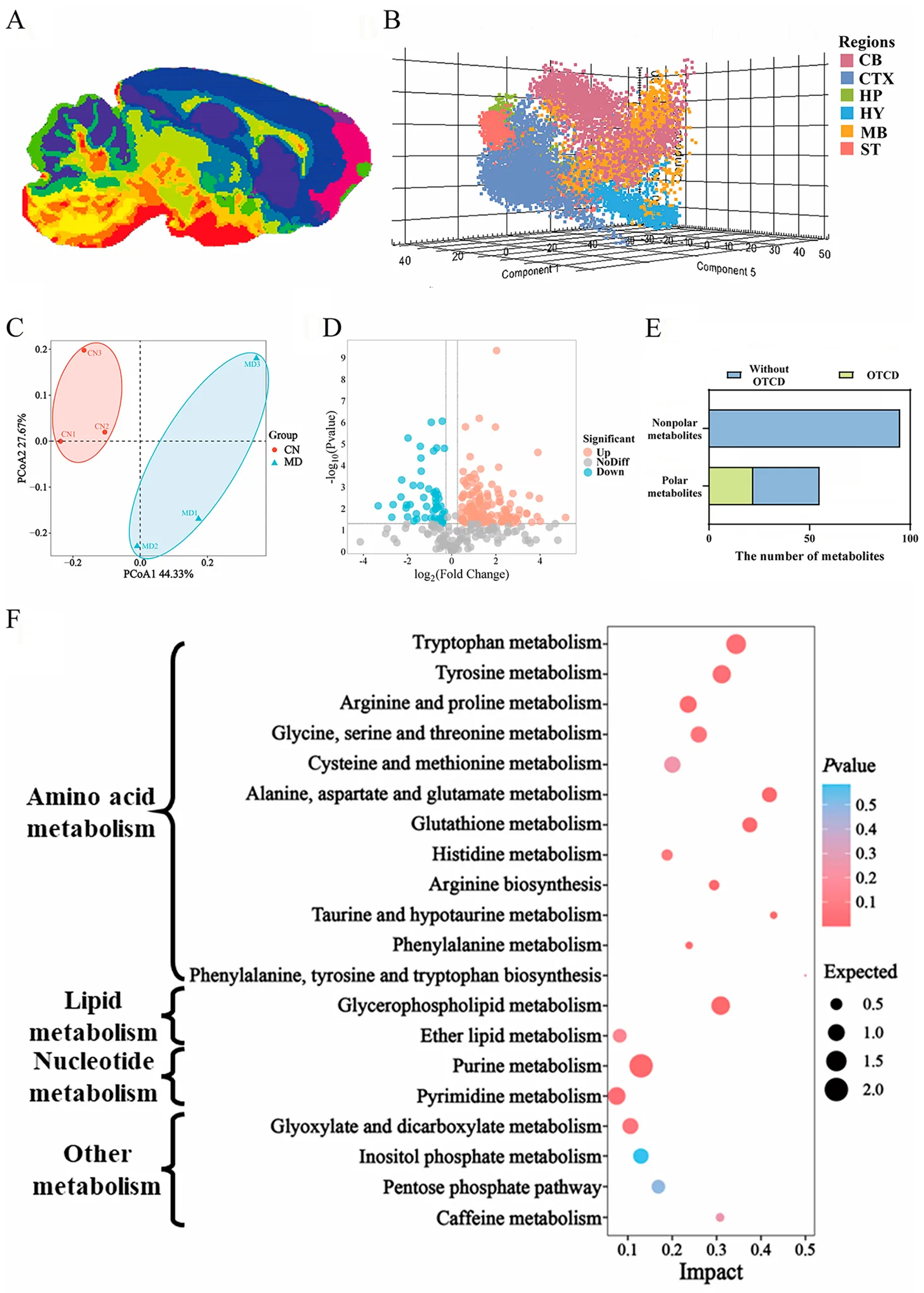
Global spatial profiling to reveal metabolite alterations in PCPA-induced insomniac rat brain. (**A**) Brian tissue section segmentation; (**B**) PCA of each microregion; (**C**) PCoA of CN and MD groups (*n* = 3); (**D**) volcano plot of changed metabolites (*p* < 0.05 and FC > 1.2 are displayed in red; *p* < 0.05 and FC < 0.83 are displayed in blue); (**E**) bar graphs showing the number of differential metabolites; (**F**) top 20 significantly altered metabolic pathways in the brain.

Subsequently, the data were imported into SCiLS software (2020b) and normalized to total ion chromatography (TIC). As shown in Figure 5A,B, the colors of the segmented microregions of the rat brain tissue were significantly different, and the PCA clearly separated each microregion from the others. The PCoA demonstrated a distinct separation of metabolic profiles between the CN and MD groups along the PCoA1 axis (Figure 5C), which indicated that brain metabolism was disturbed in insomniac rats. The volcano plot identified 160 upregulated and 68 downregulated MS features in the MD group compared to the CN group, based on fold change criteria (FC ≥ 1.2 or ≤0.83) and a significance threshold of *p* < 0.05 in Student’s *t*-tests (Figure 5D). As shown in the histogram, 150 differential metabolites, comprising 55 polar metabolites and 95 nonpolar metabolites, were identified by comparing the data with reference standards and referring to the MetaboScape^®^, HMDB and LIPID MAPS database (Figure 5E, Tables S3 and S4). As shown in Figure 5F, the top 20 KEGG enrichment pathways with significant impact values mainly included amino acid metabolism, nucleotide metabolism, lipid metabolism and so on.

### 3.6. Interruption of Amino Acid Metabolism in PCPA-Induced Insomniac Rat Brain

**Figure 6:**
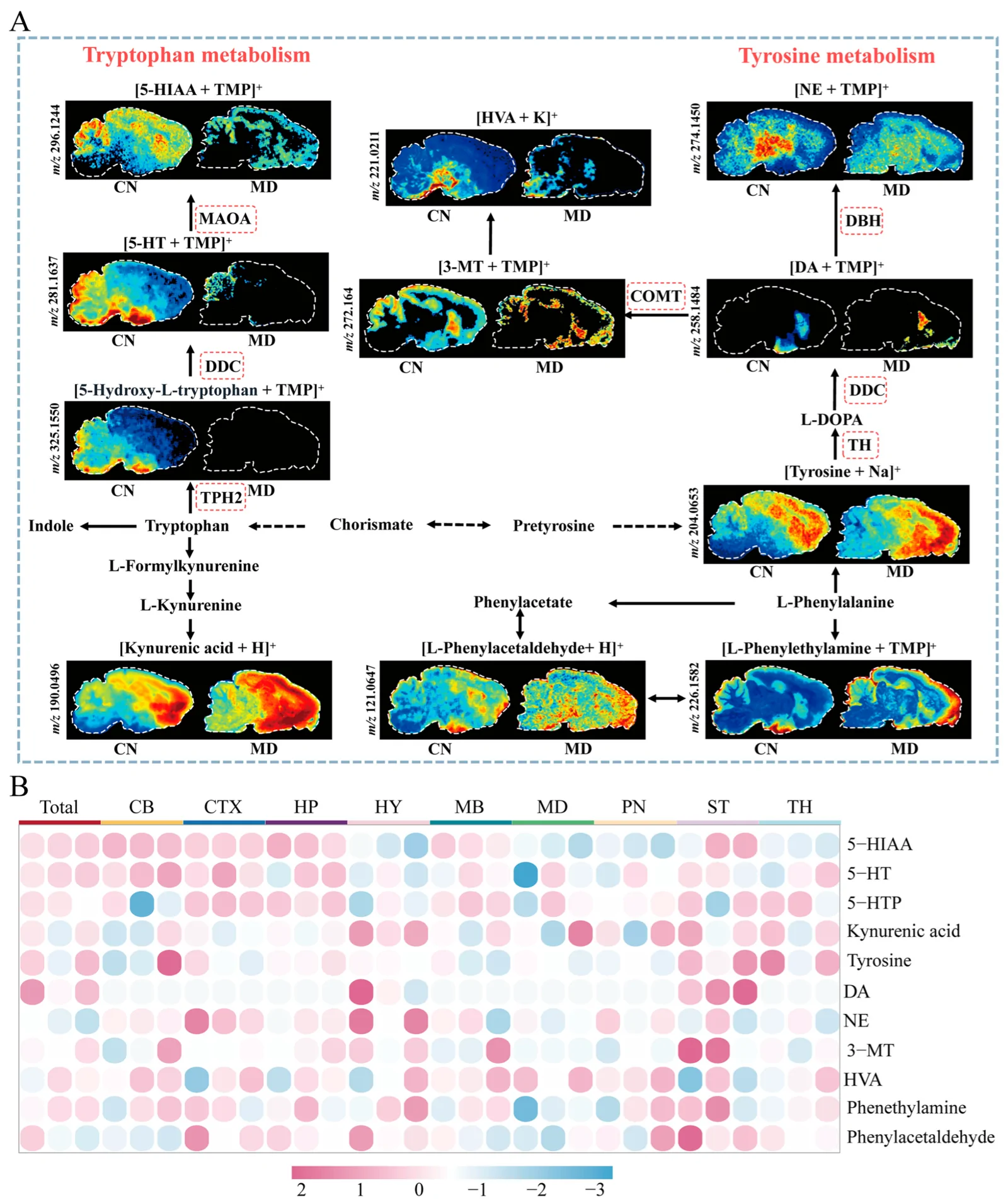
(**A**) The spatial distribution of tryptophan and tyrosine metabolic pathways. (**B**) A heat map depicting alterations in metabolite levels within brain tissue microregions in rats with PCPA-induced insomnia. Red indicates increased metabolite content and blue indicates decreased content in the MD group compared to the CN group.

A total of 43 metabolites related to amino acid metabolism were identified in the brain sections of PCPA-induced rats. Figure 6 illustrates the spatial distribution of and variations in these metabolites.

#### 3.6.1. Tryptophan Metabolism

As shown in Figure 6A, 5-HT, 5-HTP, and 5-HIAA were involved in the tryptophan metabolism pathway. Derivatized 5-HTP (325.1550) was mainly distributed in the brainstem (PN, MD and MB), CB and HY, which is consistent with 5-HT. Figure 6B illustrates that the levels of 5-HTP, 5-HT, and 5-HIAA in the MD group were markedly reduced compared to those in the CN group. Our previous studies and those of other groups have shown that the levels of 5-HT and 5-HIAA in PCPA-induced rat brains were significantly reduced when analyzed by LC-MS/MS [14,19]. Meanwhile, we identified additional downstream metabolites of 5-HT, specifically the ion (190.0496) corresponding to kynurenic acid, which exhibited distribution patterns similar to its precursor 5-HT in both CN and MD rats.

#### 3.6.2. Tyrosine Metabolism

Tyrosine (204.0653), DA, the DA metabolite 3-methoxytyramine (3-MT, 272.1640), NE, and HVA were all detected and identified in the tyrosine metabolism pathway. Tyrosine was primarily located in the CTX, HP, ST, and HY, while derivatized 3-MT was mainly found in the ST and HY (Figure 6A), aligning with findings from Esteve et al. [24] and Guo et al. [25]. Compared with the CN group, the relative abundances of tyrosine and DA of MD group rats were significantly increased (Figure 6B). Similarly to DA, 3-MT exhibited elevated concentrations in the MD group. Consequently, enhanced DA metabolism may elevate 3-MT levels.

#### 3.6.3. Alanine, Aspartate, and Glutamate Metabolism

**Figure 7:**
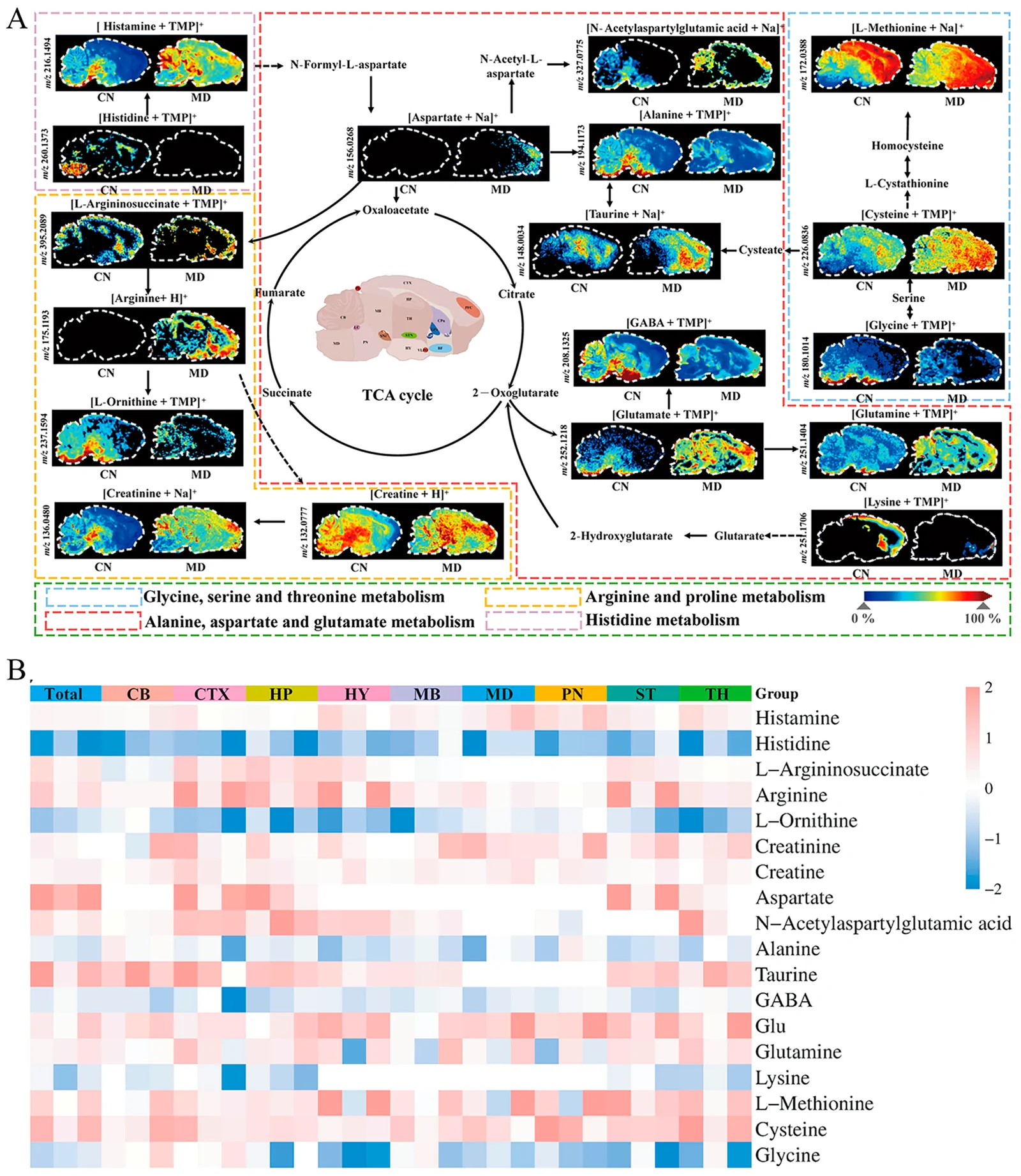
(**A**) Spatial distribution of amino acid metabolic pathways. (**B**) The heat map shows the levels of metabolite content changes in the microregions of brain tissue in rats with PCPA-induced insomnia (red represents an increase in content and blue represents a decrease in content in the MD group compared with the CN group).

As shown in Figure 7A, seven key metabolites, including aspartate (*m*/*z* 156.1494), N-acetylaspartylglutamic acid (327.0775), derivatized alanine (194.1173), taurine (148.0034), derivatized GABA (208.1325), Glu (252.1218), and glutamine (251.1404), were involved in the alanine, aspartate, and glutamate metabolism pathway. Glutamine, the precursor of Glu, was spatially resolved, and its levels were increased in the CB and CTX of insomniac rats. Studies have shown that the level of glutamine decreases during sleep and increases during wakefulness [26]. Compared with the CN group, the relative intensities of aspartate in the CTX, N-acetylaspartylglutamic acid mainly in the CB, MB and HY, and taurine in the CTX, HP and ST showed increases in MD rats (Figure 7B). Previous studies found that the level of aspartate was increased in sleep-deprived rats [27]. In addition, alanine (194.1173) was the most abundant in the brainstem (PN, MD and MB) and HY regions of the CN group but exhibited a decrease in most regions of the MD group.

Similarly, we obtained localization information for derivatized phenylethylamine (226.1582) and phenylacetaldehyde (121.0647), mainly distributed in the CTX, HP, HY and ST. Compared with the CN group, the contents of phenylethylamine and phenylacetaldehyde in the MD group were significantly increased. Previous studies have shown that phenylethylamine could promote the synthesis and release of DA and increase the excitability of the CTX, manifesting as central excitation and subsequently affecting sleep [28].

#### 3.6.4. Histidine, Arginine and Proline, and Glycine, Serine and Threonine Metabolism

Histidine is an important amino acid for protein synthesis. Histamine, a metabolite of histidine, is mainly involved in immune responses and neural regulation [29]. As shown in Figure 7A, histamine (216.1494) was predominantly present in the MB, CB, and HY, whereas derivatized histidine ion peaks (260.1373) were primarily observed in the brainstem, aligning with prior findings [30]. Histamine is an excitatory neurotransmitter produced by the tuberomammillary nucleus located in HY histaminergic neurons [31]. Elevated histamine levels can cause sleep disturbances, including trouble falling asleep, frequent awakenings, and decreased sleep efficiency [32]. As shown in Figure 7A, the MD group exhibited altered histamine metabolism in the brainstem and hypothalamus compared to the CN group, characterized by a significant increase in histamine content and a notable decrease in histamine levels.

The arginine and proline metabolism pathway involved five metabolites, including derivatized L-argininosuccinate (395.2089) and ornithine (237.1594), as well as arginine (175.1193), creatinine (136.0480) and creatine (132.0777). Arginine, one of the final products of the urea cycle, is generated by the cleavage of L-Argininosuccinate. Arginine and L-Argininosuccinate exhibited similar distribution patterns, predominantly located in the CB, CTX, HP, ST, and HY regions. The relative intensities of arginine and L-argininosuccinate were significantly increased in MD rats. Meanwhile, the derivative ornithine (237.1594) significantly decreased in the brainstem and HY of PCPA-induced insomniac rats in comparison with the CN group. Creatine is the main metabolite in energy metabolism, and creatinine is the metabolite of creatine [33]. According to Wang et al. (2023) [34], creatinine (136.0480) and creatine (132.0777) predominantly localize to the CB, brainstem, and HY regions. In MD rats, creatinine and creatine levels were significantly higher than those in the CN group, consistent with findings by Gordji-nejad et al. [35].

Glycine is an inhibitory neurotransmitter that can regulate sleep and immune responses [36]. Derivatized glycine (180.1014) was primarily found in the MD, PN, and HY regions, aligning with the distribution of the glycine receptor [34]. We found that the content of glycine in MD rats was significantly decreased. Research has shown that supplementation with glycine before bedtime can significantly improve sleep quality [37]. In MD rats, the signal intensities of derivatized methionine (172.0388) and cysteine (226.0836) were reduced in the CTX, HP, HY, and ST regions compared to CN rats.

### 3.7. Changed Microregional Levels of Metabolites in Tryptophan and Tyrosine Metabolism Detected by Targeted Metabolomics

**Figure 8:**
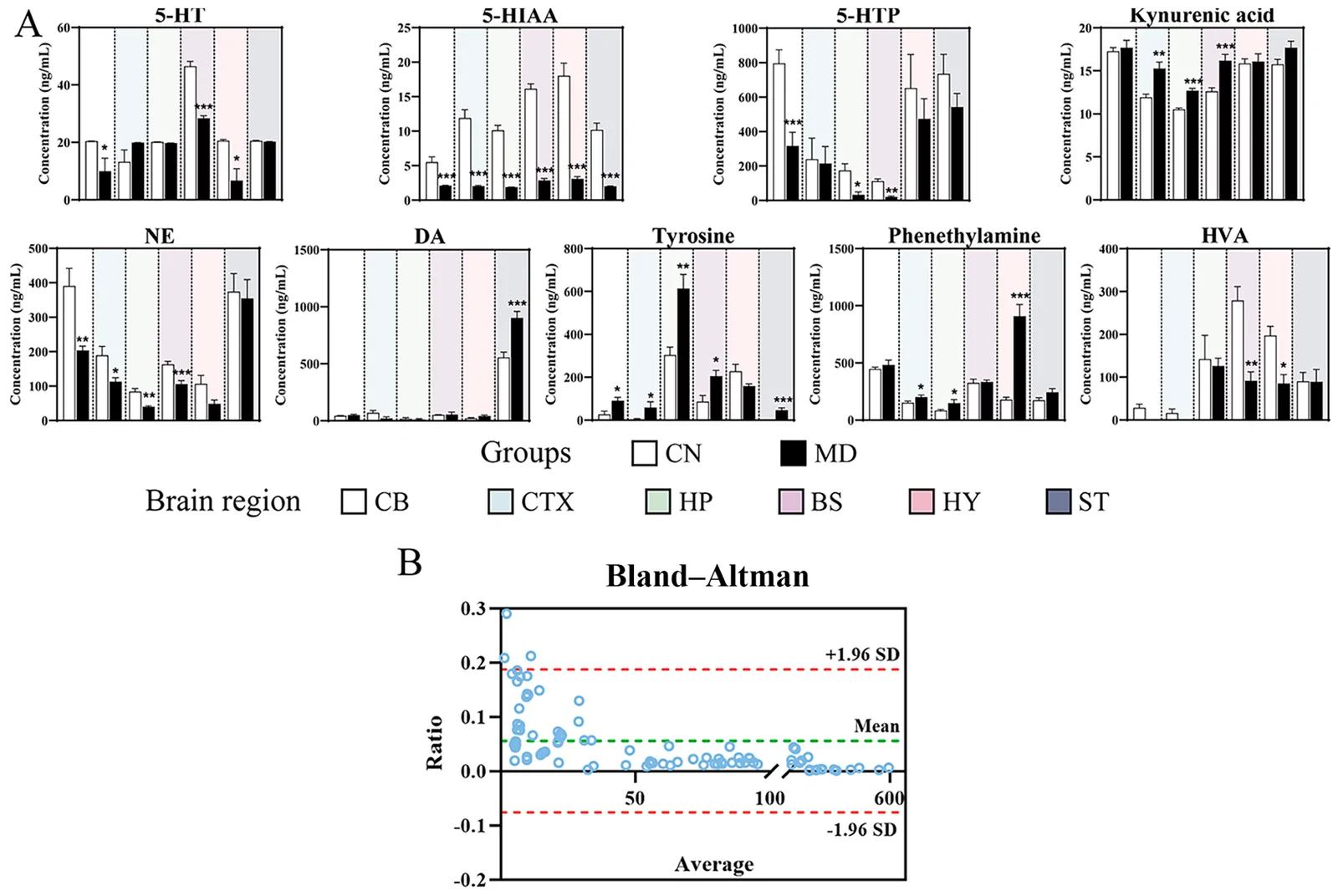
(**A**) Targeted metabolomics identified key metabolites involved in tryptophan and tyrosine metabolism across various brain regions, including the CB, CTX, HP, BS, HY, and ST (*n* = 6). Significance levels compared to the CN group: * *p* < 0.05, ** *p* < 0.01, *** *p* < 0.001; compared to the MD group: * *p* < 0.05, ** *p* < 0.01, *** *p* < 0.001. (**B**) Bland–Altman analysis of the analysis results of the MADLI-MSI technique and LC-MS/MS technique (red dotted lines: 95% limits of consistency; green dotted line: mean of the ratio).

We developed a method to quantify nine metabolites in six different brain regions (HY, ST, CTX, CB, HP, and BS) by LC-MS/MS. The analysis parameters, regression equations, linear range, and precision metrics are presented in Figure S4 and listed in Tables S5–S7. Utilizing the established analytical methods, the concentrations of the nine metabolites in the six brain microregions, including the CB, CTX, HP, brainstem, HY, and ST, were quantified. As shown in Figure 8A, both approaches exhibited similar distribution trends across various regions. The CN group exhibited significantly higher levels of 5-HT in the BS, HY, and CB; 5-HTP in the CB, HP, and BS; and 5-HIAA across all six brain regions compared to the other group. Importantly, the contents of DA and 3-MT only significantly increased in the ST of MD rats. Moreover, the content of tyrosine, the precursor of DA, displayed an increasing trend across five brain microregions, excluding the HY, of MD rats. Conversely, the NE levels in the CTX, HP, and brainstem of MD rats exhibited a significant decline. In addition, the content of HVA in the brainstem and HY of MD rats significantly decreased.

A Bland–Altman analysis was conducted to compare the results showing the nine metabolite concentrations determined by LC-MS/MS and relative signal intensities obtained by MSI in the six different regions. As illustrated in Figure 8B, the mean value of the ratio was approximately zero, and the majority of data points fell within the 95% limits of agreement, indicating consistency between the results of the two techniques. The calculation formula is as follows: $y=\log_{10}(\mathrm{MSI}\;\mathrm{value}/\mathrm{LC}\text{-}\mathrm{MS}\;\mathrm{value})$, where the MSI value represents the relative signal intensities of the nine metabolites in the six different regions. The LC-MS value represents the nine metabolite concentrations in the six different regions.

### 3.8. Validation of Key Enzymes in the Tryptophan and Tyrosine Metabolic Pathways

**Figure 9:**
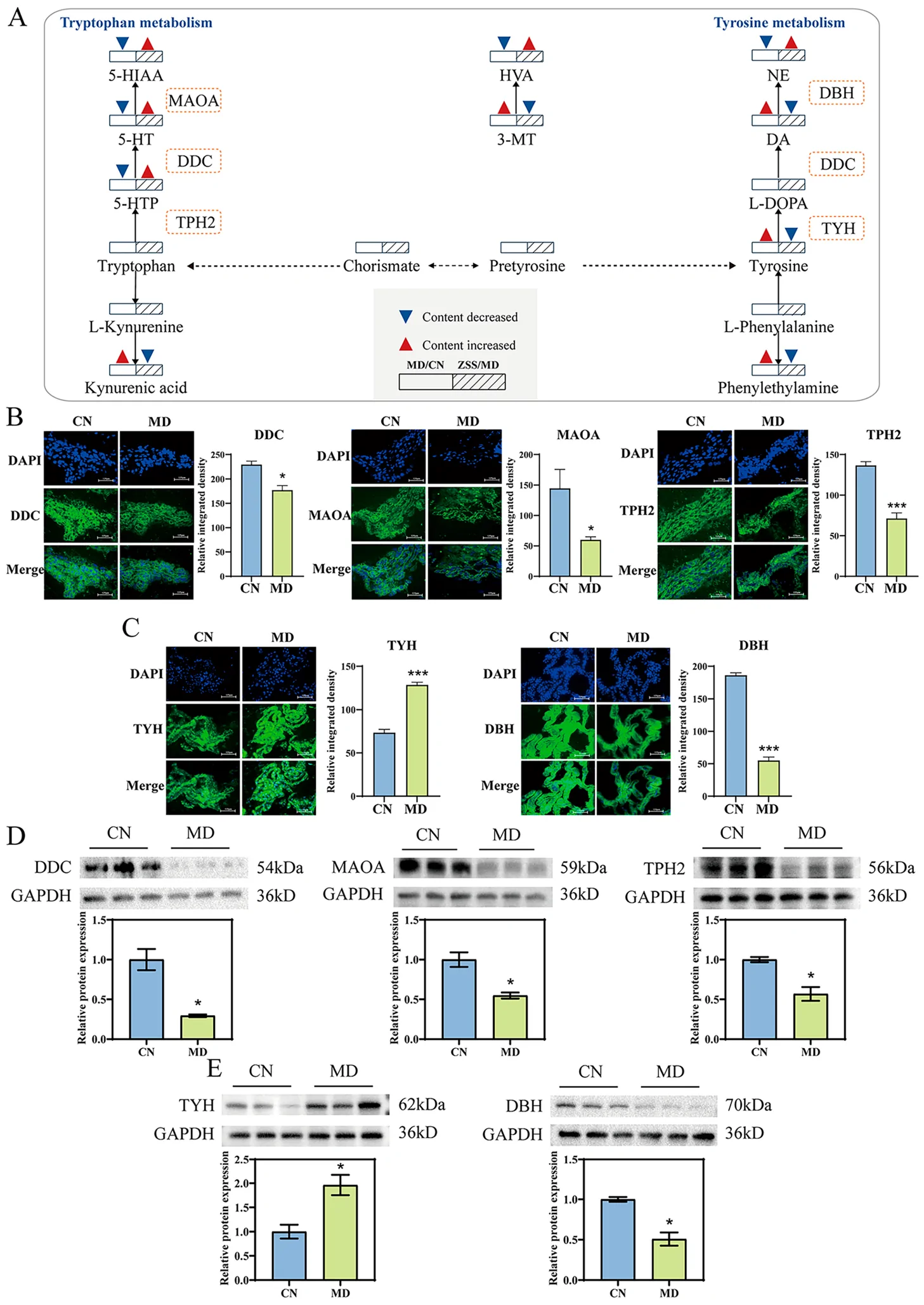
(**A**) Diagram of tryptophan and tyrosine metabolic pathways. (**B**) Immunofluorescence images of tryptophan metabolic enzymes (DOPA, MAOA, and TPH2) in brain tissue micro regions across four rat groups. (**C**) Immunofluorescence images of tyrosine metabolic enzymes (TYH and DBH) in brain tissue micro regions across four rat groups (scale = 125 μm), along with fluorescence quantitative analysis of three enzymes (*n* = 3). (**D**) PCPA altered the protein expression of tryptophan metabolism enzymes DDC, MAOA, TPH2 of CN and MD rat in brainstem. (**E**) WB analysis of levels for tyrosine metabolism enzymes TYH, and DBH of CN and MD rat in striatum. GAPDH was used as the loading control. Data are presented as mean ± SEM. Statistical significance compared to the CN group: * *p* < 0.05, *** *p* < 0.001.

As shown in Figure 9A,B, the expression levels of TPH2 and DDC in the raphe nuclei of insomniac rats were significantly reduced, which is consistent with the decreasing trend of 5-HT content in the imaging results. This localization aligns with previous findings of serotonergic innervation in the ventral striatum and projections from the dorsal raphe nucleus to the pallidum’s external and internal segments. In addition, WB analysis showed that the expression levels of the key enzymes DDC, MAOA, and TPH2 in the tryptophan metabolic pathway were significantly reduced in the brainstem and ST (*p* < 0.05) (Figure 9D). As shown in Figure 9C, compared with the CN group, the expression of TYH in the ST of MD rats was significantly increased, while the expression of DBH in the ST region of MD rats exhibited a decreasing trend, corresponding to the decreased NE content in the imaging results. Meanwhile, WB analysis verified that PCPA treatment significantly increased TYH expression (*p* < 0.05), while DBH expression decreased (Figure 9E). These results showed that the metabolic enzymes MAOA, DDC, and TPH2 within the Trp-5-HTP-5-HT-5-HIAA metabolic pathway in the brainstem and TYH and DBA within the tyr-DA-NE metabolic pathway in the ST were disturbed.

## 4. Discussion

In MALDI-MSI analysis, the matrix plays a crucial role in influencing detection sensitivity, reproducibility, resolution, and signal suppression [38]. We compared two different kinds of matrices, DHB and CHCA, and optimized the derivatization reagents and matrix spraying method for in situ brain tissue. In this study, CHCA exhibited superior performance in terms of detection intensity. Moreover, three commercially available derivatization reagents, CA, TPP-TFB and TMP-TFB, were applied to the CHCA-coated tissues for imaging analysis. The reactions of CA with neurotransmitters are condensation reactions, while the reactions of TPP-TFB and TMP-TFB with neurotransmitters are pyran salt reactions. Previous studies have reported that the aldehyde groups of CA derivatization reagents reacted with primary amines to form derivatives, which in turn enhanced the intensity of neurotransmitters and amino acid metabolites [39]. However, our study found that the TMP-TFB derivatization reagent detected the largest number of neurotransmitters with better sensitivity and higher resolution.

According to the reported complex pathogenesis of insomnia, disturbances in neurotransmitters in the brain are widely accepted to be associated with insomnia [40]. In our previous study, we found altered neurotransmitters in brain homogenate in PCPA-induced insomniac rats [19]. To analyze the changes in brain microregions’ neurotransmitter concentrations, we first detected 5-HT, GABA, Glu, Ach, DA, NE, 5-HIAA and HVA in the brain in situ using MALDI-MSI. Furthermore, spatial metabolomics and KEGG enrichment analysis showed that tryptophan and tyrosine metabolism were the major metabolic pathways. In addition, alanine, aspartate, and glutamate metabolism, histidine, arginine and proline metabolism, and glycine, serine and threonine metabolism were involved in microregion-specific neurochemical perturbations in the brain of PCPA-induced insomniac rats. Our earlier research identified a strong association between insomnia in PCPA-induced rats and the biosynthesis of phenylalanine, tyrosine, and tryptophan, as well as the metabolism of tyrosine and tryptophan [31]. Thus, we focused on tryptophan metabolism and tyrosine metabolism in nine different brain regions in PCPA-induced insomniac rats. Due to the absence of a separation technique in MSI, matrix effects and interference generally hinder result quantitation. The MSI findings were evaluated against the quantitative outcomes obtained from LC-MS/MS.

Tryptophan metabolism involves various biochemical pathways that convert tryptophan into several bioactive compounds. These pathways play crucial roles in physiological processes, including neurotransmitter synthesis, immune response modulation, and the production of metabolites like serotonin and melatonin. Understanding these pathways is essential for gaining insights into their impact on health and disease. Prior research indicated that disruptions in tryptophan metabolism impacted sleep, mood, and cognitive performance [41]. 5-HT acts as an inhibitory neurotransmitter in the brain, playing a significant role in circadian rhythms and sleep disorders, and is frequently used as an insomnia indicator [19]. The dorsal raphe nucleus in the midbrain is the primary site for 5-HT synthesis and release, playing a crucial role in sleep–wake regulation [42]. Neurons in the raphe nuclei can synthesize and release 5-HT, a key product of tryptophan metabolism [43]. In this study, the relative signal intensities of 5-HT, 5-HIAA, and 5-HTP determined by MSI and their concentrations obtained by LC-MS exhibited similar distribution trends across various regions. The CN group exhibited significantly higher levels of 5-HT in the BS, HY, and CB; 5-HTP in the CB, HP, and BS; and 5-HIAA across all six brain regions compared to the other group. The Bland–Altman analysis further confirmed the consistency between the results of the two techniques.

As we know, changes in the levels of metabolites are influenced by metabolic enzymes. TPH is an essential enzyme in the biosynthesis of 5-HT, crucial for both peripheral and central nervous system functions. TPH facilitates the transformation of tryptophan into 5-HTP, which is subsequently metabolized into 5-HT. This step was identified as the rate-limiting factor in serotonin synthesis. DDC, also referred to as aromatic L-amino acid decarboxylase, is an enzyme essential for various biological processes. It catalyzes the decarboxylation of 5-HTP to produce 5-HT, which is an essential step in the synthesis of neurotransmitters. MAOA, a mitochondrial enzyme, is crucial for the catabolism of 5-HT to 5-HIAA in the brain, significantly impacting mood regulation and behavior [44]. We found that the metabolic enzymes MAOA, DDC, and TPH2 in the brainstem were altered in PCPA-induced insomniac rats, which corresponds to the disturbed Trp-5-HTP-5-HT-5-HIAA metabolic pathway.

Tyrosine metabolism is another key disturbed metabolic pathway in the brains of rats with PCPA-induced insomnia in this study. Initial microdialysis research in rats revealed elevated extracellular dopamine levels during the dark phase compared to the light phase in regions with dopaminergic terminals [45]. An increase in tyrosine in the brain will promote the synthesis of DA, thereby affecting the quality of sleep [46]. In our research, DA was mainly distributed in ST regions of the CN group but exhibited a significant increase in the MD group. The ST is an important site for studying tyrosine metabolism and dopaminergic neural function [47]. Previous MSI studies showed that DA was mainly localized in the ST microregion in sagittal slices of the rat brain [48], and the content of DA in the brains of PCPA-induced insomniac mice was significantly increased [49]. TYH is a key enzyme, mainly involved in the synthesis of catecholamine neurotransmitters. Tyrosine is catalyzed by TYH to generate DA, and part of DA is further synthesized into NE by DBH. DBH is a key neurotransmitter synthase responsible for converting DA to NE, which is a crucial step in the catecholamine synthesis pathway. These enzymes are crucial targets for treating depression. After selective REM sleep deprivation, the activity of TYH in the rat brain was significantly increased. In this study, we also found that the gene and protein expression levels of TYH were increased, while those of DBH were decreased, corresponding to increased DA contents in the ST, further supporting that TYH and DBA within the tyr-DA-NE metabolic pathway in the ST were disturbed in insomniac rats.

There are also several limitations of this method. Future studies may apply this validated spatial metabolomics approach to evaluate the region-specific pharmacodynamic effects of sedative–hypnotic candidates.

## 5. Conclusions

In this study, we established an optimized derivatization-assisted MALDI MSI workflow to investigate the spatially resolved metabolic alterations in a PCPA-induced insomnia rat model. The current study is the first to demonstrate the distribution of eight neurotransmitters across nine brain microregions as well as the changes in and spatial distribution of small-molecule metabolites following PCPA treatment. Notably, we observed specific patterns of tyrosine and tryptophan metabolism alterations in insomniac rat brains. The results confirmed that PCPA administration markedly depleted 5-HT and its precursors and metabolites, including 5-HTP and 5-HIAA, particularly in the brainstem, cerebellum, hypothalamus, cerebral cortex, and hippocampus, which was corroborated by reduced expression of TPH2 and DDC in the brainstem. In parallel, region-specific alterations in tyrosine, DA, and NE were observed mainly in the striatum, along with the increased expression of TYH and decreased expression of DBH. This study demonstrates that region-specific alterations in tryptophan and tyrosine metabolism pathways provide mechanistic insights into insomnia pathogenesis.

## Data Availability

The original contributions presented in this study are included in the article and Supplementary Materials.

## Supplementary Materials

The following supporting information can be downloaded at https://www.mdpi.com/article/10.3390/metabo16080543/s1, Figure S1: (A) The content of 5-HT in rat serum (Compared with the CN group, **** p* < 0.001), (B) Tracing of locomotion for representative Control and Model mice. (C) Quantification of total distance, average speed, and movement time during the OFT; Figure S2: MSI visualization of sagittal facial neurotransmitters in rat brains under CHCA substrate spraying conditions (A) The number of spraying layers was 4 times and the drying time was 10 seconds; (B) The number of spray coatings is 6 times and the drying time is 30 seconds. (C) The number of spray coatings is 10 times and the drying time is 30 seconds. Detection sensitivity of the optimized spraying conditions of the matrix solution. (B) neurotransmitters; (C) The number of detected ions under the optimized conditions for matrix solution spraying; Figure S3: Investigation of the coverage rate of metabolites. (A) The number of ions detected and the number of metabolites identified; (B) Mass spectra of metabolites detected in brain tissue (*m*/*z* 50–800); Figure S4: Multi-reaction monitoring mode (MRM) chromatograms of eight metabolites and internal standard compounds in blank brain tissue (A) and blank brain tissue + reference substance + internal standard (B); Figure S5: Full membrane images for uncropped WB data (*n* = 3); Table S1: Parameters for MRM mass spectrometry acquisition; Table S2: Mass axis stability of 8 neurotransmitters detected by MSI in the same rat brain tissue section; Table S3: Polarized differential metabolites in the brain tissue of PCPA rats detected under the positive ion mode; Table S4: Non-polar differential metabolites in the brain tissue of PCPA rats detected under the positive ion mode; Table S5: Linear equations and linear ranges of 9 metabolites in brain tissue; Table S6: Precisions and accuracies for the determination of 9 metabolites from the assay sample; Table S7: Recoveries and matrix effects for the analytes in rat brain (Mean ± SD, *n* = 3).
