# Supplementary material for: Spatial Metabolomics Reveals Microregion-Specific Neurochemical Perturbations in the Brains of PCPA-Induced Insomniac Rats: Integration of MALDI-MSI and Targeted LC-MS/MS
Source: Metabolites. 2026 Jul 31;16(8):543. doi: 10.3390/metabo16080543 (PMC13515548; doi:10.3390/metabo16080543)
Supplement: Supplementary file 1 [file metabolites-16-00543-s001.zip › metabolites-4440074-supplementary.pdf]

Spatial metabolomics reveals microregion-specific neurochemical perturbations in the brain of PCPA-induced insomnia rats: integration of MALDI-MSI and targeted LC-MS/MS

Yan Yan[1,2*], Jiaying Liu[2], Yingjian Deng[2], Yu Tao[2], Xinxin Li[2], Chenhui Du[3], Kun Yang[1], Ruiping Zhang[1,4*]

1. Shanxi Bethune Hospital, Shanxi Academy of Medical Sciences, Third Hospital of Shanxi Medical University, Tongji Shanxi Hospital, Taiyuan, 030032, China.

2. Modern Research Center for Traditional Chinese Medicine, Shanxi University, Taiyuan 030006, China.

3. School of Traditional Chinese Materia Medica, Shanxi University of Chinese Medicine, Taiyuan 030619, China.

4. The Radiology Department of Shanxi Provincial People's Hospital Affiliated to Shanxi Medical University, Taiyuan, 030001, China.

*Corresponding author: Ruiping Zhang, zrp_7142@sxmu.edu.cn; Kun Yang, yk_52920@163.com

[a]These authors contribute equally to this study.

Supplementary Files

1. Targeted metabolomics

1.1 *Stock solution, calibration standards and quality control samples*

Stock solutions of 5-HIAA, kynurenic acid, 5-HT, 5-HTP, phenylalanine, tyrosine, DA, HVA and NE were prepared in water- methanol 5 : 5, *V/V*) at the concentration of 1.0 mg·mL^{-1}. The mixed stock solution containing each of the above 9 standard neurotransmitters was prepared in the final concentration range of 1-100 ng·mL^{-1} for 5-HIAA, 10-500 ng·mL^{-1} for kynurenic acid, 10-1000 ng·mL^{-1} for 5-HT, 50-1000 ng·mL^{-1} for 5-HTP, 100-1000 ng·mL^{-1} for phenylalanine, 1-1000 ng·mL^{-1} for tyrosine, 80-800 ng·mL^{-1} for DA, 25-1000 ng·mL^{-1} for HVA, 50-500 ng·mL^{-1} for NE. The quality control (QC) samples were prepared in the same way as the calibration solution.

1.2 *UPLC-ESI-MS/MS analysis*

LC-MS/MS analysis was performed using an AB SCIEX 6500 QQQ mass spectrometer connected to an LC-20A UHPLC system (Agilent. Analytes were separated on an Waters Acquity BEH amide (1.7 μm, 2.1 mm × 100 mm) at 40 °C. Mobile phase A was water containing 0.1% formic acid (v/v) and 20 mmoL·L^{-1} ammonium formate, and phase B was ACN. The gradient program was conducted as follows: 0-3.5 min, 100% B; 3.5-5 min, 100%-80% B; 5-8 min, 80%-50% B; 8-9 min, 50% -45% B; 9-10 min, 45%B; 10-10.01 min, 45%-100% B. The flow rate of the mobile phase was 0.25 mL/min. The injection volume was 5 μL.

The quantification was performed using MRM in positive ionization. The optimal MS parameters obtained were as follows: vaporizer temperature, 500 °C; nebulizing gas (GS1), 50 psi; drying gas (GS2), 50 psi; curtain gas, 35 psi. Nitrogen gas was used in all analyses. Data acquisition and processing were performed using Analyst software version 1.6.2 (AB SCIEX, Singapore).

Table S1 Parameters for MRM mass spectrometry acquisition

pathway	metabolites	Rt (min)	MRM transition	DP	CE
tryptophan	5-HIAA	1.50	192.1/146.1	45	23
	Kynurenic acid	3.43	190.1/144.0	173	26
	5-HT	6.09	177.1/160.1	100	15
	5-HTP	8.67	221.2/204.1	152	18
tyrosine	Phenylalanine	8.25	166.1/120.1	30	18

	Tyrosine	8.67	182.1/136.1	25	15
	DA	8.45	154.1/137.0	100	12
	NE	1.59	170.1/152.1	40	13
	HVA	8.52	183.1/137.1	40	15
I.S.	L-tryptophan-d3	8.29	208.1/147.1	60	35

Table S2 Mass axis stability of 8 neurotransmitters detected by MSI in the same rat brain tissue section

Analytes	*m/z*	ppm
5-HT	281.1637	±6.9
GABA	208.1325	±4.9
Glu	252.1218	±7.3
Ach	146.1173	±7.3
DA	258.1484	±4.1
NE	274.1450	±5.0
5-HIAA	296.1244	±4.4
HVA	221.0194	±8.8

Figure legends

Figure S1 (A). The content of 5-HT in rat serum (Compared with the CN group, $^{***}P < 0.001$), (B). Tracing of locomotion for representative Control and Model mice. (C). Quantification of total distance, average speed, and movement time during the OFT.

Figure S2 MSI visualization of sagittal facial neurotransmitters in rat brains under CHCA substrate spraying conditions (A): The number of spraying layers was 4 times and the drying time was 10 seconds; (B): The number of spray coatings is 6 times and the drying time is 30 seconds. (C): The number of spray coatings is 10 times and the drying time is 30 seconds. Detection sensitivity of the optimized spraying conditions of the matrix solution. (B) neurotransmitters; (C): The number of detected ions under the optimized conditions for matrix solution spraying.

Figure S3 Investigation of the coverage rate of metabolites. (A): The number of ions detected and the number of metabolites identified; (B): Mass spectra of metabolites detected in brain tissue (m/z 50-800).

Figure S4 Multi-reaction monitoring mode (MRM) chromatograms of eight metabolites and internal standard compounds in blank brain tissue (A) and blank brain tissue + reference substance + internal standard (B)

Figure S5 Full membrane images for uncropped WB data (n=3).

Figure S1

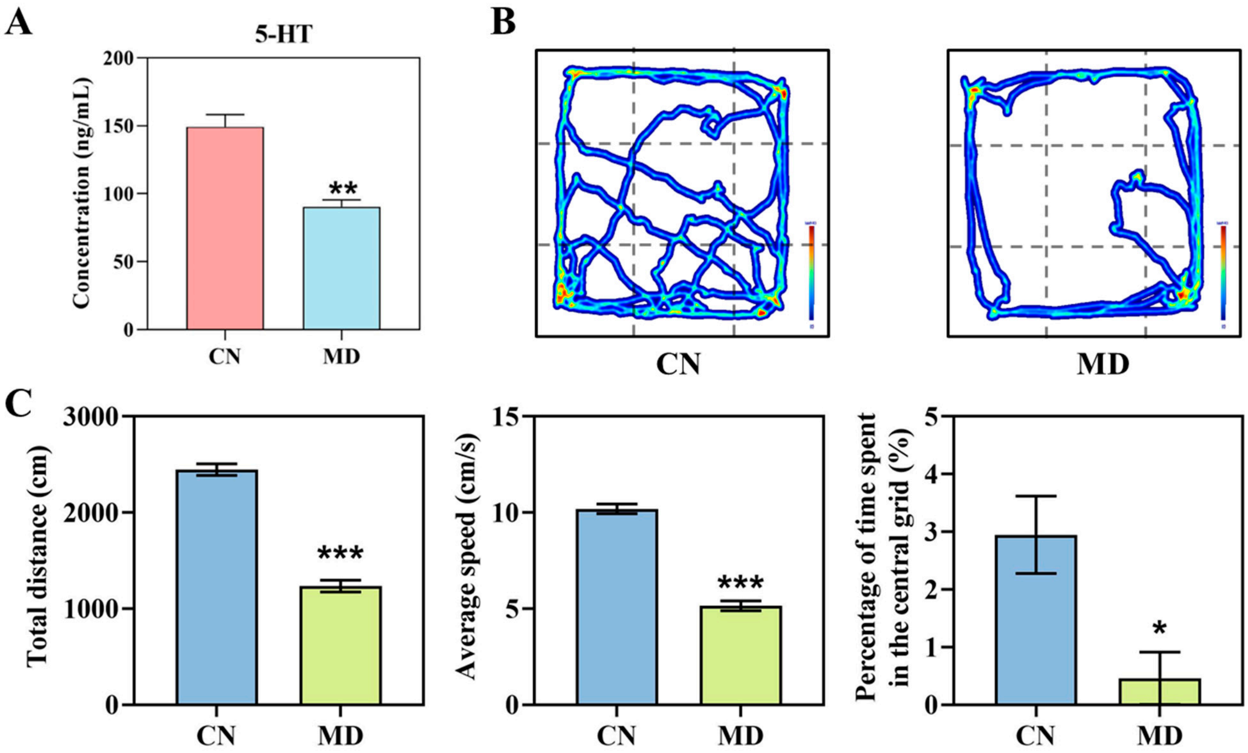
A
5-HT
Concentration (ng/mL)
200
150
100
50
0
**
CN
MD
B
CN
MD
C
Total distance (cm)
3000
2000
1000
0
***
CN
MD
Average speed (cm/s)
15
10
5
0
***
CN
MD
Percentage of time spent in the central grid (%)
5
4
3
2
1
0
*
CN
MD

Figure S2

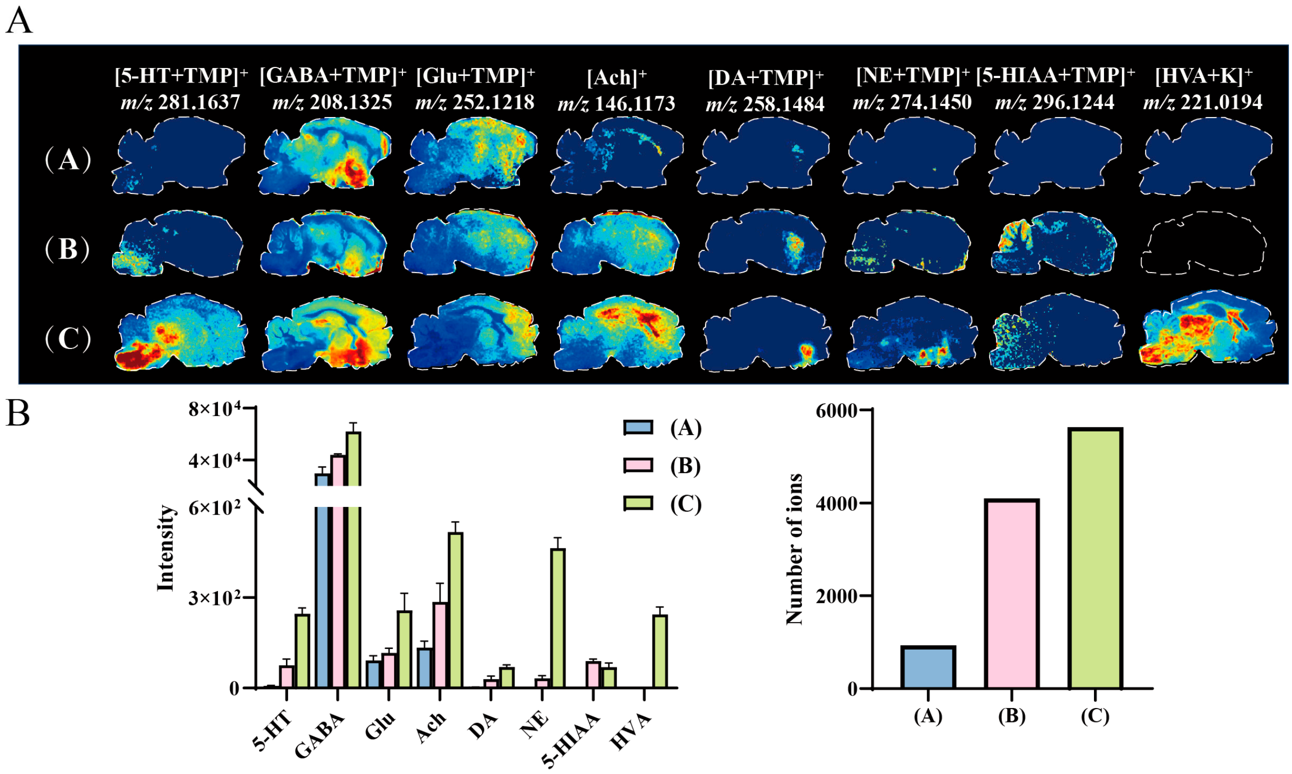
A
[5-HT+TMP]+ m/z 281.1637
[GABA+TMP]+ m/z 208.1325
[Glu+TMP]+ m/z 252.1218
[Ach]+ m/z 146.1173
[DA+TMP]+ m/z 258.1484
[NE+TMP]+ m/z 274.1450
[5-HIAA+TMP]+ m/z 296.1244
[HVA+K]+ m/z 221.0194
(A)
(B)
(C)
B
Intensity
8×10⁴
4×10⁴
6×10²
3×10²
0
5-HT
GABA
Glu
Ach
DA
NE
5-HIAA
HVA
(A)
(B)
(C)
Number of ions
6000
4000
2000
0
(A)
(B)
(C)

Figure S3

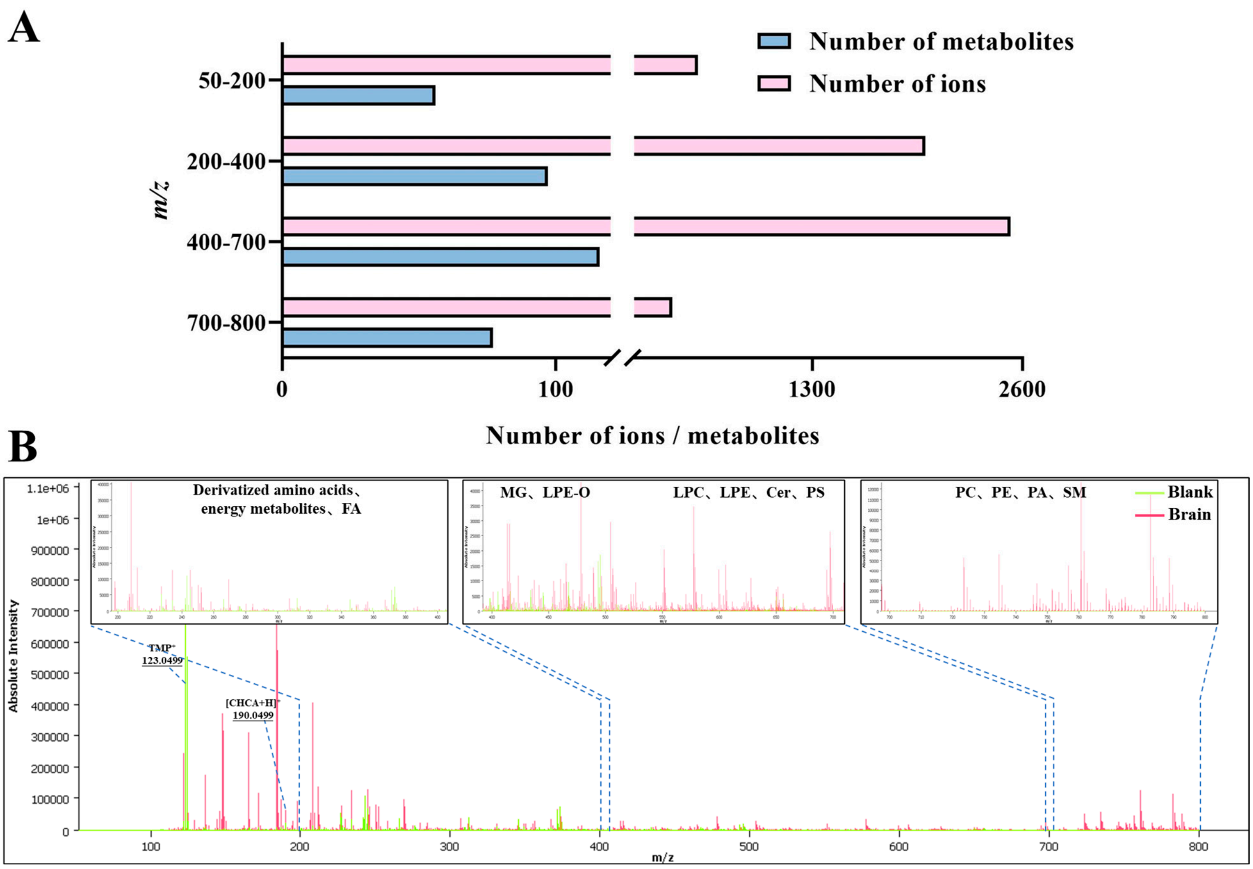
A
Number of metabolites
Number of ions
50-200
200-400
400-700
700-800
m/z
0
100
1300
2600
Number of ions / metabolites
B
Derivatized amino acids、energy metabolites、FA
MG、LPE-O
LPC、LPE、Cer、PS
PC、PE、PA、SM
Blank
Brain
TMP+ 123.0499
[CHCA+H]+ 190.0499
Absolute Intensity
m/z

Figure S4

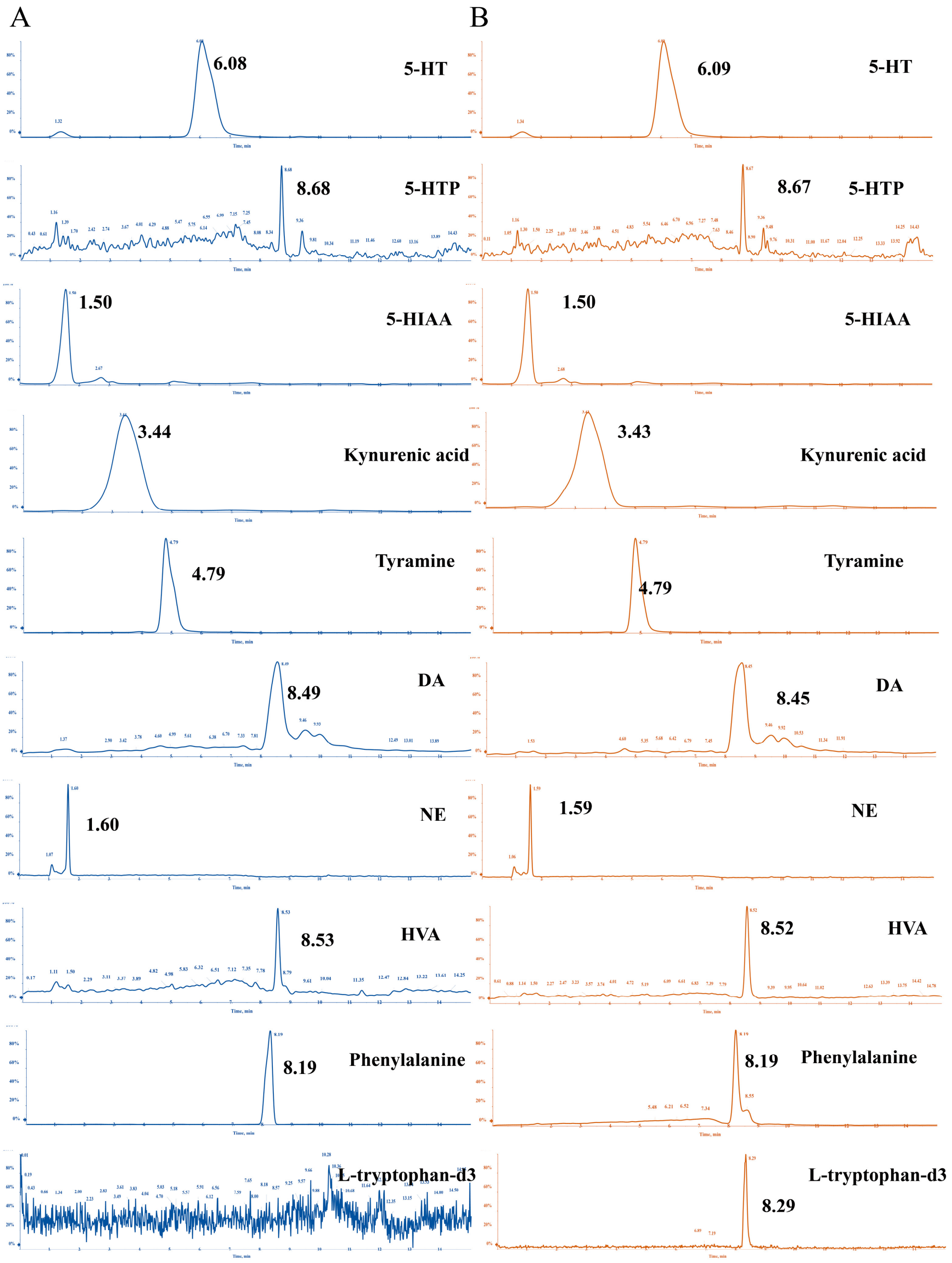
A
B
6.08
5-HT
6.09
5-HT
8.68
5-HTP
8.67
5-HTP
1.50
5-HIAA
1.50
5-HIAA
3.44
Kynurenic acid
3.43
Kynurenic acid
4.79
Tyramine
4.79
Tyramine
8.49
DA
8.45
DA
1.60
NE
1.59
NE
8.53
HVA
8.52
HVA
8.19
Phenylalanine
8.19
Phenylalanine
L-tryptophan-d3
8.29
L-tryptophan-d3

Figure S5

A

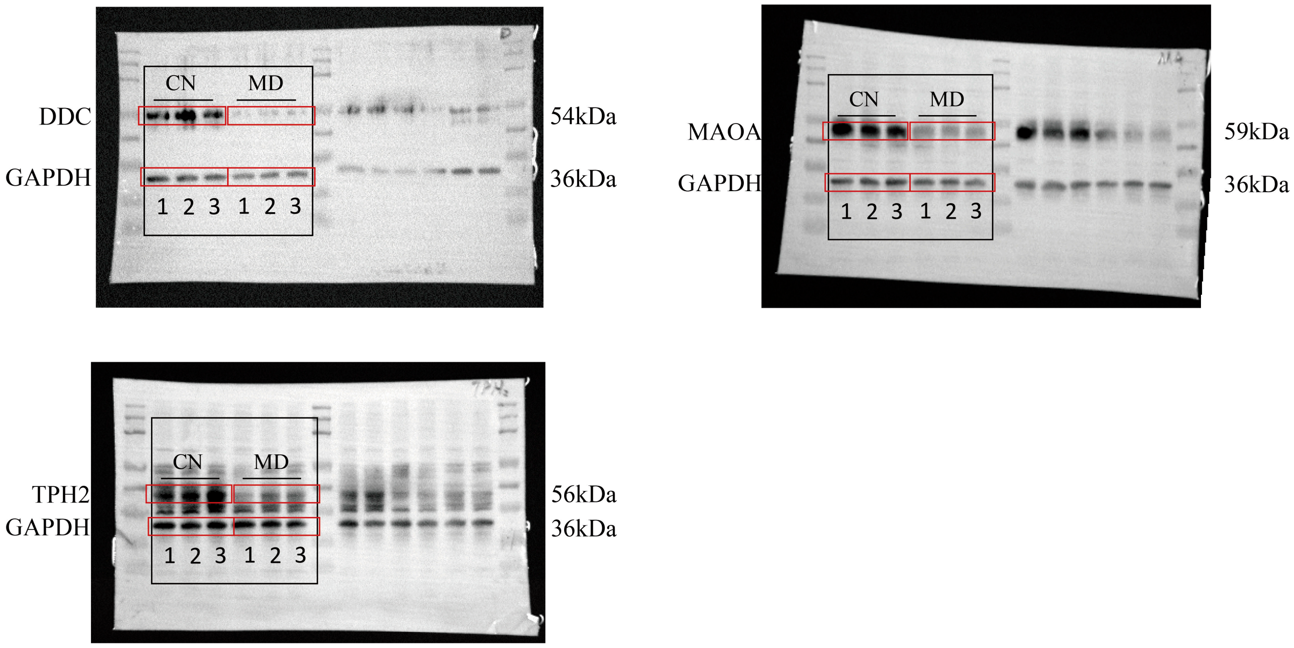
DDC
GAPDH
CN
MD
1 2 3 1 2 3
54kDa
36kDa
MAOA
GAPDH
CN
MD
1 2 3 1 2 3
59kDa
36kDa
TPH2
GAPDH
CN
MD
1 2 3 1 2 3
56kDa
36kDa

B

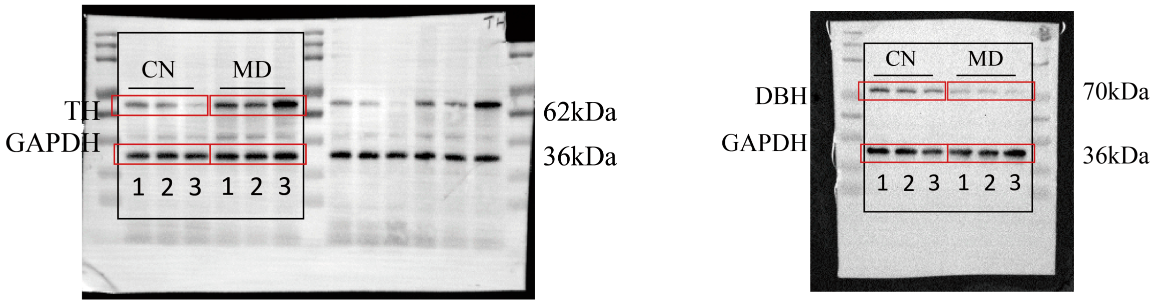
TH
GAPDH
CN
MD
1 2 3 1 2 3
62kDa
36kDa
DBH
GAPDH
CN
MD
1 2 3 1 2 3
70kDa
36kDa

Table S3 Polarized differential metabolites in the brain tissue of PCPA rats detected under the positive ion mode.

No.	Name	Formula	Adduct	Theoretical *m/z*	Experimental *m/z*	ppm	FC	*P*	Belong
1	Indoline	C_8H_9N	$[M+H]^+$	120.0808	120.0805	2.50	1.67↑	0.0004	HMDB
2	Phenylacetaldehyde	C_8H_8O	$[M+H]^+$	121.0648	121.0647	0.83	2.01↑	0.0454	HMDB
3	Phenylethyl alcohol	$C_8H_{10}O$	$[M+H]^+$	123.0804	123.0801	2.44	1.36↑	0.0039	HMDB
4	Creatine	$C_4H_9N_3O_2$	$[M+H]^+$	132.0768	132.0777	-6.81	1.63↑	0.0009	HMDB
5	Creatinine	$C_4H_7N_3O$	$[M+Na]^+$	136.0481	136.0480	0.74	1.88↑	0.0213	HMDB
6	Adenine	$C_5H_5N_5$	$[M+H]^+$	136.0618	136.0616	1.47	8.23↑	0.0197	HMDB
7	Indole-3-carboxaldehyde	C_9H_7NO	$[M+H]^+$	146.0600	146.0600	0.00	3.40↑	0.0471	HMDB
8	Ach*	$C_7H_{16}NO_2$	$[M]^+$	146.1176	146.1173	2.05	0.56↓	0.0128	Standard
9	Taurine	$C_2H_7NO_3S$	$[M+Na]^+$	148.0039	148.0034	3.38	6.73↑	0.0487	HMDB
10	3-Methylhistamine	$C_6H_{11}N_3$	$[M+Na]^+$	148.0845	148.0840	3.38	0.30↓	0.0381	HMDB
11	Valine	$C_5H_{11}NO_2$	$[M+K]^+$	156.0421	156.0436	-9.61	5.07↑	0.0287	HMDB
12	Indole-3-carboxylic acid	$C_9H_7NO_2$	$[M+H]^+$	162.0550	162.0549	0.62	4.53↑	0.0032	HMDB
13	Carnitine*	$C_7H_{15}NO_3$	$[M+H]^+$	162.1125	162.1124	0.62	2.40↑	0.0009	Standard
14	Monoethanolamine	C_2H_7NO	$[M+TMP]^+$	166.1226	166.1224	1.20	0.62↓	0.0001	HMDB
15	Phenyllactate	$C_9H_{10}O_3$	$[M+TMP]^+$	167.0703	167.0691	7.18	3.18↑	0.0176	HMDB
16	N-Methyl-aspartate	$C_5H_9NO_4$	$[M+Na]^+$	170.0424	170.0419	2.94	3.47↑	0.0199	HMDB
17	L-Methionine*	$C_5H_{11}NO_2S$	$[M+Na]^+$	172.0403	172.0388	8.72	2.74↑	0.0452	Standard
18	Arginine*	$C_6H_{14}N_4O_2$	$[M+H]^+$	175.1190	175.1193	-1.71	2.82↑	0.0214	Standard
19	Glycine*	$C_2H_5NO_2$	$[M+TMP]^+$	180.1019	180.1014	2.78	0.45↓	0.0025	Standard
20	3-Methyladenine	$C_6H_7N_5$	$[M+K]^+$	188.0333	188.0344	-5.85	5.65↑	0.0261	HMDB
21	Kynurenic acid*	$C_{10}H_7NO_3$	$[M+H]^+$	190.0499	190.0496	1.58	2.32↑	0.0424	Standard
22	Alanine*	$C_3H_7NO_2$	$[M+TMP]^+$	194.1176	194.1173	1.55	0.53↓	0.0033	Standard
23	N-Acetylhistidine	$C_8H_{11}N_3O_3$	$[M+H]^+$	198.0873	198.0887	-7.07	1.41↑	0.0325	HMDB
24	myo-Inositol	$C_6H_{12}O_6$	$[M+Na]^+$	203.0526	203.0526	0.00	4.37↑	0.0001	HMDB
25	Tyrosine*	$C_9H_{11}NO_3$	$[M+Na]^+$	204.0631	204.0651	-9.80	5.51↑	0.0388	Standard
26	N-lactoyl-Leucine	$C_9H_{17}NO_4$	$[M+H]^+$	204.1230	204.1229	0.49	2.36↑	0.0039	HMDB

27	GABA*	$C_4H_9NO_2$	$[M+TMP]^+$	208.1332	208.1325	3.36	0.64↓	0.0044	Standard
28	Histamine*	$C_5H_9N_3$	$[M+TMP]^+$	216.1495	216.1494	0.46	1.60↑	0.0001	Standard
29	HVA*	$C_9H_{10}0_4$	$[M+K]^+$	221.0211	221.0193	8.14	6.75↑	0.0245	Standard
30	Cysteine	$C_3H_7NO_2S$	$[M+TMP]^+$	226.0896	226.0874	9.73	5.13↑	0.0236	HMDB
31	N1-Acetylspermidine	$C_9H_{21}N_3O$	$[M+K]^+$	226.1316	226.1294	9.73	10.56↑	0.0178	HMDB
32	Phenethylamine	$C_8H_{11}N$	$[M+TMP]^+$	226.1590	226.1582	3.54	4.78↑	0.0002	HMDB
33	Ornithine	$C_5H_{12}N_2O_2$	$[M+TMP]^+$	237.1598	237.1594	1.69	0.34↓	0.0049	HMDB
34	Tyramine*	$C_8H_{11}NO$	$[M+TMP]^+$	242.1539	242.1536	1.24	1.56↑	0.0012	Standard
35	Uridine	$C_9H_{12}N_2O_6$	$[M+H]^+$	245.0768	245.0792	-9.79	9.92↑	0.0175	Standard
36	Glutamine*	$C_5H_{10}N_2O_3$	$[M+TMP]^+$	251.1390	251.1404	-5.57	1.90↑	0.0030	Standard
37	Lysine*	$C_6H_{14}N_2O_2$	$[M+TMP]^+$	251.1754	251.1731	9.16	0.44↓	0.0473	Standard
38	Glu*	$C_5H_9NO_4$	$[M+TMP]^+$	252.1230	252.1218	4.76	3.00↑	0.0419	Standard
39	DA*	$C_8H_{11}NO_2$	$[M+TMP]^+$	258.1489	258.1484	1.94	3.47↑	0.0405	Standard
40	Histidine	$C_6H_9N_3O_2$	$[M+TMP]^+$	260.1394	260.1373	8.07	0.12↓	0.0096	HMDB
41	3-MT	$C_9H_{13}NO_2$	$[M+TMP]^+$	272.1645	272.1640	1.84	2.27↑	0.0324	HMDB
42	NE*	$C_8H_{11}NO_3$	$[M+TMP]^+$	274.1438	274.1450	-4.38	0.21↓	0.0139	Standard
43	5-HT*	$C_{10}H_{12}N_2O$	$[M+TMP]^+$	281.1648	281.1637	3.91	0.58↓	0.0233	Standard
44	Guanosine	$C_{10}H_{13}N_5O_5$	$[M+H]^+$	284.0989	284.0969	7.04	5.28↑	0.0156	HMDB
45	Adenosine*	$C_{10}H_{13}N_5O_4$	$[M+Na]^+$	290.0860	290.0845	5.17	4.78↑	0.0419	Standard
46	5-HIAA*	$C_{10}H_9NO_3$	$[M+TMP]^+$	296.1281	296.1266	5.07	0.42↓	0.0011	Standard
47	dCMP	$C_9H_{14}N_3O_7P$	$[M+H]^+$	308.0642	308.0654	-3.90	1.93↑	0.0006	HMDB
48	Glutathione	$C_{10}H_{17}N_3O_6S$	$[M+H]^+$	308.0911	308.0924	-4.22	5.21↑	0.0108	HMDB
49	5-HTP*	$C_{11}H_{12}N_2O_3$	$[M+TMP]^+$	325.1547	325.1550	-0.92	0.66↓	0.0055	Standard
50	N-Acetylaspartylglutamic acid	$C_{11}H_{16}N_2O_8$	$[M+Na]^+$	327.0799	327.0775	7.34	2.22↑	0.0118	HMDB
51	CMP	$C_9H_{14}N_3O_8P$	$[M+K]^+$	362.0150	362.0128	6.08	3.53↑	0.0008	HMDB
52	dGMP	$C_{10}H_{14}N_5O_7P$	$[M+Na]^+$	370.0523	370.0533	-2.70	8.35↑	0.0116	HMDB
53	Adenosine 5'-phosphate disodium	$C_{10}H_{12}N_5Na_2O_7P$	$[M+H]^+$	392.0343	392.0334	2.30	4.40↑	0.0048	HMDB
54	L-Argininosuccinate	$C_{10}H_{18}N_4O_6$	$[M+TMP]^+$	395.1925	395.1902	5.82	2.23↑	0.0158	HMDB
55	GMP	$C_{10}H_{14}N_5O_8P$	$[M+TMP]^+$	468.1279	468.1293	-2.99	2.08↑	0.0364	HMDB

Table S4 Non-polar differential metabolites in the brain tissue of PCPA rats detected under the positive ion mode.

No.	Name	Formula	Adduct	Theoretical *m/z*	Experimental *m/z*	Ppm	FC	*P*	Belong
1	Propionic acid	C3H6O2	[M+K]+	112.9999	112.9994	4.42	6.60↑	0.0393	Lipid maps
2	FA 8:3	$C_8H_{10}O_2$	$[M+H]^+$	139.0754	139.0752	-1.44	4.95↑	0.0324	Lipid maps
3	FA 9:6	$C_9H_6O_2$	$[M+H]^+$	147.0441	147.0434	-4.76	6.07↑	0.0240	Lipid maps
4	FA 6:2	$C_6H_8O_2$	$[M+K]^+$	151.0156	151.0151	-3.31	7.66↑	0.0293	Lipid maps
5	FA 8:6	$C_8H_4O_2$	$[M+Na]^+$	155.0104	155.0101	-1.94	6.10↑	0.0493	Lipid maps
6	FA 7:3	$C_7H_8O_2$	$[M+K]^+$	163.0156	163.0154	-1.23	2.63↑	0.0124	Lipid maps
7	FA 7:2	$C_7H_{10}O_2$	$[M+K]^+$	165.0312	165.0309	-1.82	5.84↑	0.0459	Lipid maps
8	Phosphocholine	$C_5H_{14}NO_4P$	$[M+H]^+$	184.0733	184.0730	1.63	2.57↑	0.0049	Lipid maps
9	Propenoylcarnitine	$C_{10}H_{17}NO_4$	$[M+H]^+$	216.1230	216.1245	-6.94	2.66↑	0.0202	Lipid maps
10	FA 13:4	$C_{13}H_{18}O_2$	$[M+Na]^+$	229.1199	229.1220	9.17	2.37↑	0.0054	Lipid maps
11	FA 16:6	$C_{16}H_{20}O_2$	$[M+H]^+$	245.1536	245.1529	-2.86	1.52↑	0.0413	Lipid maps
12	Glycerophosphocholine	$C_8H_{20}NO_6P$	$[M+H]^+$	258.1101	258.1097	1.55	4.15↑	0.0217	Lipid maps
13	FA 19:6	$C_{19}H_{26}O_2$	$[M+Na]^+$	309.1825	309.1842	5.50	3.54↑	0.0439	Lipid maps
14	3-oxo-nonadecanoic acid	$C_{19}H_{36}O_3$	$[M+H]^+$	313.2737	313.2737	0.00	2.04↑	0.0003	Lipid maps
15	FA 20:4	$C_{20}H_{32}O_2$	$[M+Na]^+$	327.2295	327.2264	-9.47	5.43↑	0.0121	Lipid maps
16	DHA	$C_{22}H_{32}O_2$	$[M+H]^+$	329.2475	329.2443	9.72	0.24↓	0.0095	Lipid maps
17	Sphingosine 1-phosphate (d16:1-P)	$C_{16}H_{34}NO_5P$	$[M+K]^+$	390.1806	390.1776	7.69	3.24↑	0.0491	Lipid maps
18	PC (16:0/14:0)	$C_{38}H_{76}NO_8P$	$[M+H]^+$	706.5381	706.5343	-5.38	0.42↓	0.0031	Lipid maps
19	PC (O-16:0/16:1)	$C_{40}H_{80}NO_7P$	$[M+H]^+$	718.5745	718.5726	-2.64	2.98↑	0.0073	Lipid maps
20	PC 32:5	$C_{40}H_{70}NO_8P$	$[M+H]^+$	724.4912	724.4964	7.18	0.57↓	0.0000	Lipid maps
21	PC 30:1	$C_{38}H_{74}NO_8P$	$[M+Na]^+$	726.5044	726.5078	4.68	0.62↓	0.0000	Lipid maps
22	PC (14:0/16:0)	$C_{38}H_{76}NO_8P$	$[M+Na]^+$	728.5201	728.5176	-3.43	0.60↓	0.0035	Lipid maps
23	PC (18:1/14:0)	$C_{40}H_{78}NO_8P$	$[M+H]^+$	732.5538	732.5556	2.46	0.53↓	0.0278	Lipid maps

24	PC (O-18:1/16:0)	$C_{42}H_{84}NO_7P$	$[M+H]^+$	746.6058	746.6035	-3.08	2.35↑	0.0020	Lipid maps
25	PC (18:0/14:0)	$C_{40}H_{80}NO_8P$	$[M+Na]^+$	756.5514	756.5505	-1.19	0.68↓	0.0281	Lipid maps
26	PC (18:1/16:1)	$C_{42}H_{80}NO_8P$	$[M+H]^+$	758.5694	758.5628	-8.70	0.60↓	0.0021	Lipid maps
27	PC O-36:6	$C_{44}H_{78}NO_7P$	$[M+H]^+$	764.5589	764.5533	-7.32	1.54↑	0.0000	Lipid maps
28	PC (18:5/18:5)	$C_{44}H_{68}NO_8P$	$[M+H]^+$	770.4755	770.4804	6.36	1.38↑	0.0029	Lipid maps
29	PC (P-18:0/18:0)	$C_{44}H_{88}NO_7P$	$[M+H]^+$	774.6371	774.6368	-0.39	2.93↑	0.0293	Lipid maps
30	PC (16:0/18:1)	$C_{42}H_{82}NO_8P$	$[M+Na]^+$	782.5670	782.5668	-0.26	0.66↓	0.0091	Lipid maps
31	PC 34:0	$C_{42}H_{84}NO_8P$	$[M+Na]^+$	784.5827	784.5766	-7.77	0.80↓	0.0000	Lipid maps
32	PC (22:2/14:0)	$C_{44}H_{84}NO_8P$	$[M+H]^+$	786.6007	786.5956	-6.48	0.57↓	0.0018	Lipid maps
33	PC (22:1/14:0)	$C_{44}H_{86}NO_8P$	$[M+H]^+$	788.6164	788.6158	-0.76	0.62↓	0.0079	Lipid maps
34	LPC 16:0	$C_{24}H_{50}NO_7P$	$[M+H]^+$	496.3398	496.3391	-1.41	1.43↑	0.0002	Lipid maps
35	LPC O-18:4	$C_{26}H_{48}NO_6P$	$[M+H]^+$	502.3292	502.3246	-9.16	1.60↑	0.0196	Lipid maps
36	LPC O-18:3	$C_{26}H_{50}NO_6P$	$[M+H]^+$	504.3449	504.3444	-0.99	1.40↑	0.0312	Lipid maps
37	LPC P-18:1	$C_{26}H_{52}NO_6P$	$[M+H]^+$	506.3605	506.3595	-1.97	1.74↑	0.0023	Lipid maps
38	LPC P-18:0	$C_{26}H_{54}NO_6P$	$[M+H]^+$	508.3762	508.3787	4.92	2.28↑	0.0256	Lipid maps
39	LPC 18:2	$C_{26}H_{50}NO_7P$	$[M+H]^+$	520.3398	520.3402	0.77	1.35↑	0.030	Lipid maps
40	LPC 18:1	$C_{26}H_{52}NO_7P$	$[M+H]^+$	522.3554	522.3547	-1.34	1.39↑	0.0032	Lipid maps
41	LPC 18:0	$C_{26}H_{54}NO_7P$	$[M+H]^+$	524.3711	524.3697	-2.67	1.44↑	0.0005	Lipid maps
42	LPC 20:3	$C_{28}H_{52}NO_7P$	$[M+H]^+$	568.3374	568.3322	-9.15	1.75↑	0.0128	Lipid maps
43	LPC 20:1	$C_{28}H_{56}NO_7P$	$[M+H]^+$	588.3426	588.3455	4.93	1.93↑	0.0401	Lipid maps
44	LPC 22:6	$C_{30}H_{50}NO_7P$	$[M+H]^+$	590.3217	590.3224	1.19	0.37↓	0.0000	Lipid maps
45	LPC 22:0	$C_{30}H_{62}NO_7P$	$[M+H]^+$	618.3896	618.3916	3.23	2.31↑	0.0483	Lipid maps
46	PE 19:0	$C_{24}H_{48}NO_8P$	$[M+H]^+$	510.3190	510.3220	5.88	14.95↑	0.0000	Lipid maps
47	PE 28:2	$C_{33}H_{62}NO_8P$	$[M+H]^+$	654.4105	654.4102	-0.46	0.38↓	0.0002	Lipid maps
48	PE 34:0	$C_{39}H_{78}NO_8P$	$[M+H]^+$	720.5538	720.5560	3.05	0.30↓	0.0008	Lipid maps
49	PE 36:1	$C_{41}H_{80}NO_8P$	$[M+Na]^+$	768.5514	768.5547	4.29	0.69↓	0.0322	Lipid maps
50	PE 38:2	$C_{43}H_{82}NO_8P$	$[M+H]^+$	772.5851	772.5867	2.07	1.61↑	0.0130	Lipid maps
51	PE 38:1	$C_{43}H_{84}NO_8P$	$[M+H]^+$	774.6007	774.6019	1.55	0.76↓	0.0178	Lipid maps
52	LPE O-14:3	$C_{19}H_{36}NO_6P$	$[M+H]^+$	406.2353	406.2371	4.43	2.40↑	0.0034	Lipid maps

53	LPE O-17:2	$C_{22}H_{44}NO_6P$	$[M+H]^+$	450.2979	450.2980	0.22	2.26↑	0.0360	Lipid maps
54	LPE O-19:0	$C_{24}H_{52}NO_6P$	$[M+H]^+$	482.3605	482.3594	-2.28	3.71↑	0.0468	Lipid maps
55	LPE O-20:2	$C_{25}H_{50}NO_6P$	$[M+H]^+$	492.3449	492.3449	0.00	1.93↑	0.0115	Lipid maps
56	LPE 20:0	$C_{25}H_{52}NO_7P$	$[M+H]^+$	510.3554	510.3539	-2.94	1.92↑	0.0082	Lipid maps
57	LPE 22:3	$C_{27}H_{50}NO_7P$	$[M+H]^+$	532.3398	532.3424	4.88	2.04↑	0.0238	Lipid maps
58	LPE 22:6	$C_{27}H_{44}NO_7P$	$[M+Na]^+$	548.2748	548.2784	6.57	0.33↓	0.0175	Lipid maps
59	LPE 24:4	$C_{29}H_{52}NO_7P$	$[M+H]^+$	558.3554	558.3591	6.63	2.38↑	0.00010.0	Lipid maps
60	Cer 30:0;O4	$C_{30}H_{61}NO_5$	$[M+H]^+$	516.4623	516.4579	-8.52	0.43↓	0.0268	Lipid maps
61	Cer 33:4;O2	$C_{33}H_{59}NO_3$	$[M+H]^+$	518.4568	518.4591	4.44	0.59↓	0.0393	Lipid maps
62	CerP 28:1;O2	$C_{28}H_{56}NO_6P$	$[M+H]^+$	534.3918	534.3865	-9.92	4.09↑	0.0000	Lipid maps
63	CerP 30:7;O2	$C_{30}H_{48}NO_6P$	$[M+H]^+$	550.3292	550.3293	0.18	1.69↑	0.0087	Lipid maps
64	CerP 30:1;O2	$C_{30}H_{60}NO_6P$	$[M+H]^+$	562.4231	562.4175	-9.96	1.88↑	0.0292	Lipid maps
65	Cer 34:0;O4	$C_{34}H_{69}NO_5$	$[M+H]^+$	572.5249	572.5267	3.14	1.87↑	0.0222	Lipid maps
66	Cer 36:3;O3	$C_{36}H_{67}NO_4$	$[M+H]^+$	578.5142	578.5122	-3.46	0.71↓	0.0077	Lipid maps
67	Cer 36:2;O3	$C_{36}H_{69}NO_4$	$[M+H]^+$	580.5299	580.5346	8.10	1.82↑	0.0079	Lipid maps
68	CerP 32:1;O2	$C_{32}H_{64}NO_6P$	$[M+H]^+$	590.4544	590.4526	-3.05	4.77↑	0.0420	Lipid maps
69	Cer 40:5;O3	$C_{40}H_{71}NO_4$	$[M+H]^+$	630.5456	630.548	3.81	1.59↑	0.0123	Lipid maps
70	Cer 42:8;O3	$C_{42}H_{69}NO_4$	$[M+H]^+$	652.5299	652.5363	9.81	1.54↑	0.0479	Lipid maps
71	Cer 44:5;O2	$C_{44}H_{79}NO_3$	$[M+H]^+$	670.6133	670.6094	-5.82	4.33↑	0.0020	Lipid maps
72	CerP 40:7;O2	$C_{40}H_{68}NO_6P$	$[M+H]^+$	690.4857	690.4881	3.48	2.11↑	0.0125	Lipid maps
73	CerP 40:5;O2	$C_{40}H_{72}NO_6P$	$[M+H]^+$	694.5170	694.5154	-2.30	0.80↓	0.0145	Lipid maps
74	CerP 40:4;O2	$C_{40}H_{74}NO_6P$	$[M+H]^+$	696.5327	696.5302	-3.59	0.74↓	0.0000	Lipid maps
75	HexCer 34:4;O3	$C_{40}H_{71}NO_9$	$[M+H]^+$	710.5202	710.5144	-8.16	1.78↑	0.0057	Lipid maps
76	CerP 42:8;O2	$C_{42}H_{70}NO_6P$	$[M+H]^+$	716.5014	716.4996	-2.51	1.78↑	0.0000	Lipid maps
77	PA (16:0/16:0)	$C_{35}H_{69}O_8P$	$[M+Na]^+$	671.4622	671.4582	-5.96	3.15↑	0.0041	Lipid maps
78	PA (22:2/13:0)	$C_{38}H_{71}O_8P$	$[M+H]^+$	687.4959	687.4914	-6.55	0.20↓	0.0029	Lipid maps
79	PA (18:0/20:5)	$C_{41}H_{71}O_8P$	$[M+H]^+$	723.4959	723.4937	-3.04	0.76↓	0.0491	Lipid maps
80	PA (16:0/22:4)	$C_{41}H_{73}O_8P$	$[M+H]^+$	725.5116	725.5141	3.45	0.67↓	0.0034	Lipid maps
81	PA (22:6/22:4)	$C_{47}H_{73}O_8P$	$[M+H]^+$	797.5116	797.5099	-2.13	0.75↓	0.0464	Lipid maps

82	SM 26:5;O2	$C_{31}H_{55}N_2O_6P$	$[M+H]^+$	583.3871	583.3860	-1.89	1.98↑	0.0095	Lipid maps
83	SM 30:2;O2	$C_{35}H_{69}N_2O_6P$	$[M+H]^+$	645.4966	645.4949	-2.63	1.42↑	0.0295	Lipid maps
84	SM 30:0;O2	$C_{35}H_{73}N_2O_6P$	$[M+H]^+$	649.5279	649.5256	-3.54	1.80↑	0.0133	Lipid maps
85	SM 32:1;O3	$C_{37}H_{75}N_2O_7P$	$[M+H]^+$	691.5385	691.5350	-5.06	3.54↑	0.0066	Lipid maps
86	SM (d18:0/16:1)	$C_{39}H_{79}N_2O_6P$	$[M+H]^+$	703.5749	703.5713	-5.12	6.28↑	0.0060	Lipid maps
87	SM (d18:1/18:1)	$C_{41}H_{81}N_2O_6P$	$[M+H]^+$	729.5905	729.5875	-4.11	0.15↓	0.0254	Lipid maps
88	SM (d18:0/18:1)	$C_{41}H_{83}N_2O_6P$	$[M+H]^+$	731.6062	731.6057	-0.68	0.38↓	0.0000	Lipid maps
89	SM 38:7;O3	$C_{43}H_{75}N_2O_7P$	$[M+H]^+$	763.5384	763.5323	-7.99	0.77↓	0.0280	Lipid maps
90	SM 40:7;O3	$C_{45}H_{79}N_2O_7P$	$[M+H]^+$	791.5698	791.5727	3.66	0.62↓	0.0016	Lipid maps
91	PI 30:3	$C_{39}H_{69}O_{13}P$	$[M+NH_4]^+$	794.4814	794.4891	9.69	2.42↑	0.0213	Lipid maps
92	MG 20:3	$C_{23}H_{40}O_4$	$[M+NH_4]^+$	398.3265	398.3265	0.00	1.87↑	0.0024	Lipid maps
93	MG 20:2	$C_{23}H_{42}O_4$	$[M+NH_4]^+$	400.3421	400.3421	0.00	3.26↑	0.0245	Lipid maps
94	MG 22:3	$C_{25}H_{44}O_4$	$[M+NH_4]^+$	426.3578	426.3570	-1.88	2.56↑	0.0222	Lipid maps
95	MG 22:2	$C_{25}H_{46}O_4$	$[M+NH_4]^+$	428.3734	428.3693	-9.57	5.57↑	0.0169	Lipid maps

Table S5 Linear equations and linear ranges of 9 metabolites in brain tissue.

No.	Metabolites	Regression equations	r^2	Linear rang (ng/mL)
1	5-HT	$y = 0.1348x - 2.6623$	0.9912	10–1000
2	5-HTP	$y = 0.0001x + 0.0111$	0.9939	50–1000
3	5-HIAA	$y = 0.481x - 0.6285$	0.9967	1-100
4	Kynurenic acid	$y = 0.0142x - 0.1632$	0.9997	10–500
5	Tyrosine	$y = 0.014x + 3.4781$	0.9976	1–1000
6	DA	$y = 0.0043x - 0.0756$	0.9905	80–800
7	NE	$y = 0.0035x - 0.0377$	0.9918	50–500
8	HVA	$y = 0.0011x + 0.3228$	0.9963	25–1000
9	Phenylalanine	$y = 0.5049x - 19.37$	0.9926	100-1000

Table S6 Precisions and accuracies for the determination of 9 metabolites from the assay sample.

Metabolite	Nominal conc. (ng/ml)	Inter-day			Intra-day		
		Observed concentration (ng/ml)	Precision (RSD, %)	Accuracy (RE, %)	Observed concentration (ng/ml)	Precision (RSD, %)	Accuracy (RE, %)
5-HT	10.0	11.30 ± 0.17	1.54	12.97	11.65 ± 0.83	7.15	13.41
	20.0	19.95 ± 0.51	2.54	-0.24	20.26 ± 1.82	9.00	-12.37
	500.0	508.75 ± 2.65	0.52	1.75	503.63 ± 43.92	8.72	-5.25
	800.0	784.36 ± 17.09	2.18	-1.96	759.26 ± 35.38	4.66	-7.45
5-HTP	50.00	50.63 ± 0.61	1.20	1.26	48.27 ± 5.75	11.92	-10.49
	100.00	102.21 ± 0.13	0.13	2.21	99.66 ± 9.51	9.54	-12.73
	500.00	489.09 ± 12.58	0.82	-2.18	503.12 ± 48.30	9.60	2.21
	800.00	811.06 ± 10.54	1.30	1.38	796.06 ± 88.12	11.07	-1.40
5-HIAA	1.00	1.14 ± 0.06	5.15	14.97	1.15 ± 0.17	1.50	14.17
	2.00	1.83 ± 0.05	2.67	-8.58	2.01 ± 0.06	3.05	0.80
	50.00	47.34 ± 1.15	2.43	-5.32	49.74 ± 1.44	2.90	-1.96
	80.00	76.61 ± 1.86	2.45	-4.24	76.03 ± 2.53	3.33	-6.05
Kynurenic acid	10.00	9.98 ± 0.26	2.65	-0.15	10.12 ± 0.49	4.88	0.86
	20.00	20.44 ± 0.21	1.04	2.20	19.82 ± 2.20	11.11	-5.12
	250.00	257.05 ± 6.12	2.38	2.82	255.34 ± 33.19	13.00	4.08
	400.00	388.16 ± 10.91	2.81	-2.96	385.99 ± 7.14	1.85	-7.11
Tyrosine	1.00	1.13 ± 0.01	1.04	13.33	0.97 ± 0.03	3.35	-9.65
	2.00	2.14 ± 0.04	1.80	6.87	1.91 ± 0.06	1.73	-3.25
	500.00	507.81 ± 14.57	2.87	1.56	496.18 ± 22.33	4.50	-1.71

	800.00	778.89 ± 10.13	1.30	-2.64	768.98 ± 43.06	5.60	-8.70
DA	80.00	82.83 ± 0.85	1.03	3.53	85.73 ± 5.27	6.15	9.02
	160.00	163.35 ± 3.59	2.20	2.09	164.10 ± 13.36	8.14	3.39
	400.00	386.59 ± 2.71	0.70	-3.35	387.78 ± 44.48	11.47	1.96
	800.00	798.99 ± 11.59	1.45	-0.13	790.79 ± 51.64	6.35	-6.56
NE	50.00	47.98 ± 0.77	1.60	-4.04	46.94 ± 1.47	3.14	-7.05
	100.00	112.93 ± 2.81	2.49	12.93	115.59 ± 6.20	5.36	14.62
	250.00	242.66 ± 5.36	2.21	-2.94	243.96 ± 4.32	1.77	10.60
	400.00	396.93 ± 5.56	1.40	-0.77	391.16 ± 15.09	3.86	-4.51
HVA	25.00	25.28 ± 0.33	1.30	1.12	24.31 ± 2.43	9.98	-1.48
	50.00	47.67 ± 0.68	1.42	-4.65	51.26 ± 0.93	1.82	0.81
	500.00	518.74 ± 13.44	2.59	3.75	510.09 ± 36.52	7.16	-0.70
	800.00	790.89 ± 10.28	2.60	-1.14	805.50 ± 26.82	3.33	2.54
Phenylalanine	100.00	112.77 ± 1.39	1.23	12.77	113.51 ± 1.87	1.65	13.51
	200.00	217.94 ± 11.11	5.1	8.97	218.46 ± 4.68	2.14	9.23
	500.00	469.40 ± 9.39	2.0	-6.12	523.30 ± 8.90	1.70	4.66
	800.00	815.20 ± 56.25	6.9	1.90	816.08 ± 12.57	1.54	2.01

Table S7 Recoveries and matrix effects for the analytes in rat brain (Mean ± SD, *n* = 3)

Metabolite	Theoretical concentration (ng/ml)	Recovery (%)	Matrix Effect (%)
5-HT	10.00	80 ± 15.01	103 ± 8.25
	20.00	94 ± 9.19	111 ± 15.75
	500.00	89 ± 8.06	114 ± 14.92
	800.00	77 ± 13.02	112 ± 13.25
5-HTP	50.00	85 ± 8.76	76 ± 10.75
	100.00	90 ± 10.07	96 ± 17.58
	500.00	81 ± 7.92	117 ± 3.52
	800.00	77 ± 8.23	101 ± 8.60
5-HIAA	1.00	80 ± 9.64	102 ± 16.75
	2.00	86 ± 7.08	101 ± 11.55
	50.00	69 ± 9.27	111 ± 14.61
	80.00	78 ± 13.13	110 ± 5.57
Kynurenic acid	10.00	75 ± 16.85	97 ± 7.99
	20.00	99 ± 17.32	96 ± 11.56
	250.00	74 ± 18.41	87 ± 14.56
	400.00	83 ± 8.69	105 ± 16.23
Tyrosine	1.00	79 ± 9.42	97 ± 10.71
	2.00	76 ± 12.86	111 ± 11.77
	500.00	80 ± 7.27	78 ± 12.84
	800.00	72 ± 10.31	109 ± 8.23
DA	80.00	75 ± 10.41	95 ± 5.92
	160.00	67 ± 13.91	86 ± 8.03
	400.00	80 ± 12.96	99 ± 6.95
	800.00	92 ± 14.40	102 ± 9.83
NE	50.00	85 ± 9.25	115 ± 10.02
	100.00	84 ± 11.58	113 ± 8.87
	250.00	96 ± 13.73	112 ± 14.19
	400.00	84 ± 9.51	110 ± 8.67
HVA	25.00	94 ± 8.25	76 ± 5.15

	50.00	89 ± 18.67	78 ± 7.59
	500.00	82 ± 10.70	103 ± 8.24
	800.00	76 ± 9.03	87 ± 12.47
Phenylalanine	100.00	70 ± 7.72	103.± 12.66
	200.00	68 ± 12.93	104 ± 9.37
	500.00	72 ± 6.41	101 ± 5.84
	800.00	76 ± 4.63	107 ± 8.36
L-tryptophan-d3	2000.00	98 ± 6.53	105 ± 13.45
